# Deer management generally reduces densities of nymphal *Ixodes scapularis*, but not prevalence of infection with *Borrelia burgdorferi* sensu stricto

**DOI:** 10.1016/j.ttbdis.2023.102202

**Published:** 2023-05-25

**Authors:** Alynn M. Martin, Danielle Buttke, Jordan Raphael, Kelsey Taylor, Sarah Maes, Christina M. Parise, Howard S. Ginsberg, Paul C. Cross

**Affiliations:** a Caesar Kleberg Wildlife Research Institute, Texas A&M University - Kingsville, Kingsville, TX, 78363, USA; b US Geological Survey, Northern Rocky Mountain Science Center, 2327 University Way, Suite #2, Bozeman, MT, 59715 USA; c US National Park Service, Fort Collins, CO, 80525 USA; d US National Park Service, Fire Island National Seashore, Patchogue, NY, 11772 USA; e Division of Vector-Borne Diseases, National Center for Emerging and Zoonotic Infectious Diseases, Centers for Disease Control and Prevention, Fort Collins, CO 80521; f U.S. Geological Survey, Eastern Ecological Science Center, Woodward Hall-PSE, University of Rhode Island, Kingston, RI, 02881 USA

**Keywords:** Lyme disease, *Borrelia burgdorferi* sensu stricto, *Ixodes scapularis*, blacklegged tick, white-tailed deer density, density of infected nymphs

## Abstract

Human Lyme disease–primarily caused by the bacterium *Borrelia burgdorferi* sensu stricto (s.s.) in North America–is the most common vector-borne disease in the United States. Research on risk mitigation strategies during the last three decades has emphasized methods to reduce densities of the primary vector in eastern North America, the blacklegged tick (*Ixodes scapularis*). Controlling white-tailed deer populations has been considered a potential method for reducing tick densities, as white-tailed deer are important hosts for blacklegged tick reproduction. However, the feasibility and efficacy of white-tailed deer management to impact acarological risk of encountering infected ticks (namely, density of host-seeking infected nymphs; DIN) is unclear. We investigated the effect of white-tailed deer density and management on the density of host-seeking nymphs and *B. burgdorferi* s.s. infection prevalence using surveillance data from eight national parks and park regions in the eastern United States from 2014–2022. We found that deer density was significantly positively correlated with the density of nymphs (nymph density increased by 49% with a 1 standard deviation increase in deer density) but was not strongly correlated with the prevalence of *B. burgdorferi* s.s. infection in nymphal ticks. Further, while white-tailed deer reduction efforts were followed by a decrease in the density of *I. scapularis* nymphs in parks, deer removal had variable effects on *B. burgdorferi* s.s. infection prevalence, with some parks experiencing slight declines and others slight increases in prevalence. Our findings suggest that managing white-tailed deer densities alone may not be effective in reducing DIN in all situations but may be a useful tool when implemented in integrated management regimes.

## 1. Introduction

Human Lyme disease–also called Lyme borreliosis–is the most commonly reported vector-borne disease in the United States, accounting for >80% of tick-borne illness cases documented from 2004 to 2016 (Rosenberg et al., 2018). Annual Lyme disease cases alone are estimated to be over 400,000 (Hinckley et al., 2014; Kugeler et al., 2021). Lyme disease is caused by spirochetes in the *Borrelia burgdorferi* sensu lato (s.l.) complex, with *B. burgdorferi* sensu stricto (s.s.) the primary causative agent of Lyme disease in the United States (Schwartz et al., 2017). The primary vectors of *B. burgdorferi* s.s. are *Ixodes scapularis* (the blacklegged or deer tick) in the eastern and central U.S., and *Ixodes pacificus* (the western blacklegged tick) in the west. The highest incidence of Lyme disease is observed in the northeast, mid-Atlantic, and Midwest in the contiguous United States, with geographic expansion observed in the periphery of these regions (Kugeler et al., 2015).

There is widespread interest in understanding tick management practices that reduce human risk of acquiring Lyme disease, but the complex ecology, with multiple tick host species and variable environmental conditions, make it difficult to evaluate the efficacy of different interventions. *Ixodes scapularis* has a two-year life cycle with three active life stages–larva, nymph, and adult–and each life stage requires a bloodmeal. The immature stages–larval and nymphal–often feed on small mammal hosts, which can serve as reservoirs for *B. burgdorferi* s.s. (e.g., the white-footed mouse [*Peromyscus leucopus*]; Donahue et al., 1987). Human infections are often attributed to bites by the nymphal stage of *I. scapularis* owing to their small size, which makes them difficult to detect, and their potential to have been infected with *B. burgdorferi* s.s. during their first bloodmeal as larvae (Piesman and Sinsky, 1988). Attempts to reduce infection rates or densities of host-seeking *B. burgdorferi* s.s.-infected *I. scapularis* nymphs through vaccination and acaricidal treatment of important hosts for the immature stages–namely, the white-footed mouse–have shown promise with recent treatment advancements, but have financial and logistical limitations (Richer et al., 2014; Eisen, 2023).

Other control measures have focused on disrupting the reproductive life stage of *I. scapularis* to reduce the number of host-seeking nymphs on the landscape. White-tailed deer (*Odocoileus virginianus*) are the preferred hosts for adult *I. scapularis* and play an important role in the reproductive processes, providing females mating access to male *I. scapularis* as well as a bloodmeal for egg production (Watson and Anderson, 1976; Wilson et al., 1988). Interestingly, white-tailed deer are considered unable to serve as reservoirs for *B. burgdorferi* s.s. (Telford et al., 1988) and may clear infections in *Ixodes* ticks that feed on them (Roome et al., 2017). Thus, while higher deer densities may promote large larval loads in the landscape, the impact on pathogen carriage in nymphs is likely determined by larval host-use (i.e, larvae use of *B. burgdorferi* s.s. competent hosts or not). Hypotheses have emerged regarding the potential role of deer in reducing infection prevalence in nymphal ticks (the ‘dilution effect’; Schmidt and Ostfeld, 2001); however, evidence for this is inconsistent and situational (Huang et al., 2019; Goethert et al., 2022). These findings have complicated what “best practices” should be regarding deer management in the context of Lyme disease risk mitigation.

White-tailed deer reduction as a tick management strategy is controversial. Complete deer elimination has shown some success, with immature *I. scapularis* life stages eradicated three years post-deer elimination (Rand et al., 2004). However, programs that lower deer densities but do not achieve elimination have had variable effects on host-seeking *I. scapularis* nymph densities (see review by Kugeler et al., 2016). Some experts maintain that managing deer density is one of few feasible strategies for long-term management of *I. scapularis* nymph density in the landscape (Telford, 2017). Empirical evidence suggests that, while abundances of host-seeking immature ticks may experience large annual fluctuations, mean abundance can be reduced through deer reduction management (Deblinger et al., 1993). However, the required deer density threshold to successfully reduce densities of host-seeking nymphs is unclear, though 3–5 deer per km^2^ is often cited (Telford, 2017), with some empirical support (Kilpatrick et al., 2014). Other deer management efforts have achieved reduced questing nymph densities at deer densities above this threshold (5–20 deer per km^2^), and further reduced risk using integrated methods (Williams et al., 2017). However, questions remain regarding the feasibility and generalizability of deer management and density thresholds as effective ways to reduce blacklegged tick nymph densities, as this type of management can be logistically costly (cost of approximately $600 USD per deer for removal by sharpshooting; Trapp, 2012). Foundational studies citing the utility of white-tailed deer management in controlling *I. scapularis* nymph densities often occurred on small scales–both geographically (e.g., islands) and temporally (Wilson et al., 1984, Wilson et al., 1988; Deblinger et al., 1993; Rand et al., 2004)–limiting inferences regarding practical application at larger geographic scales and temporal spans.

Here, we use nine years of *I. scapularis* nymph surveillance data, *B. burgdorferi* s.s. infection prevalence data, and deer density data from eight national parks across the eastern United States to address the influence of deer density, and the efficacy of deer management, on impacting *I. scapularis* nymph densities and *B. burgdorferi* s.s. infection rates. Specifically, we aim to: (i) quantify the effect of deer density on the density of *I. scapularis* nymphs and the density of *B. burgdorferi* s.s.-infected, host-seeking nymphs in the landscape, and (ii) assess if deer management efforts yield widely applicable results across a broad, geographic scale. We utilize four hypothesis-driven statistical models in a Bayesian framework to address these aims.

## 2. Materials and Methods

### 2.1. Transect surveys, nymph densities, and pathogen testing

**Fig. 1.**
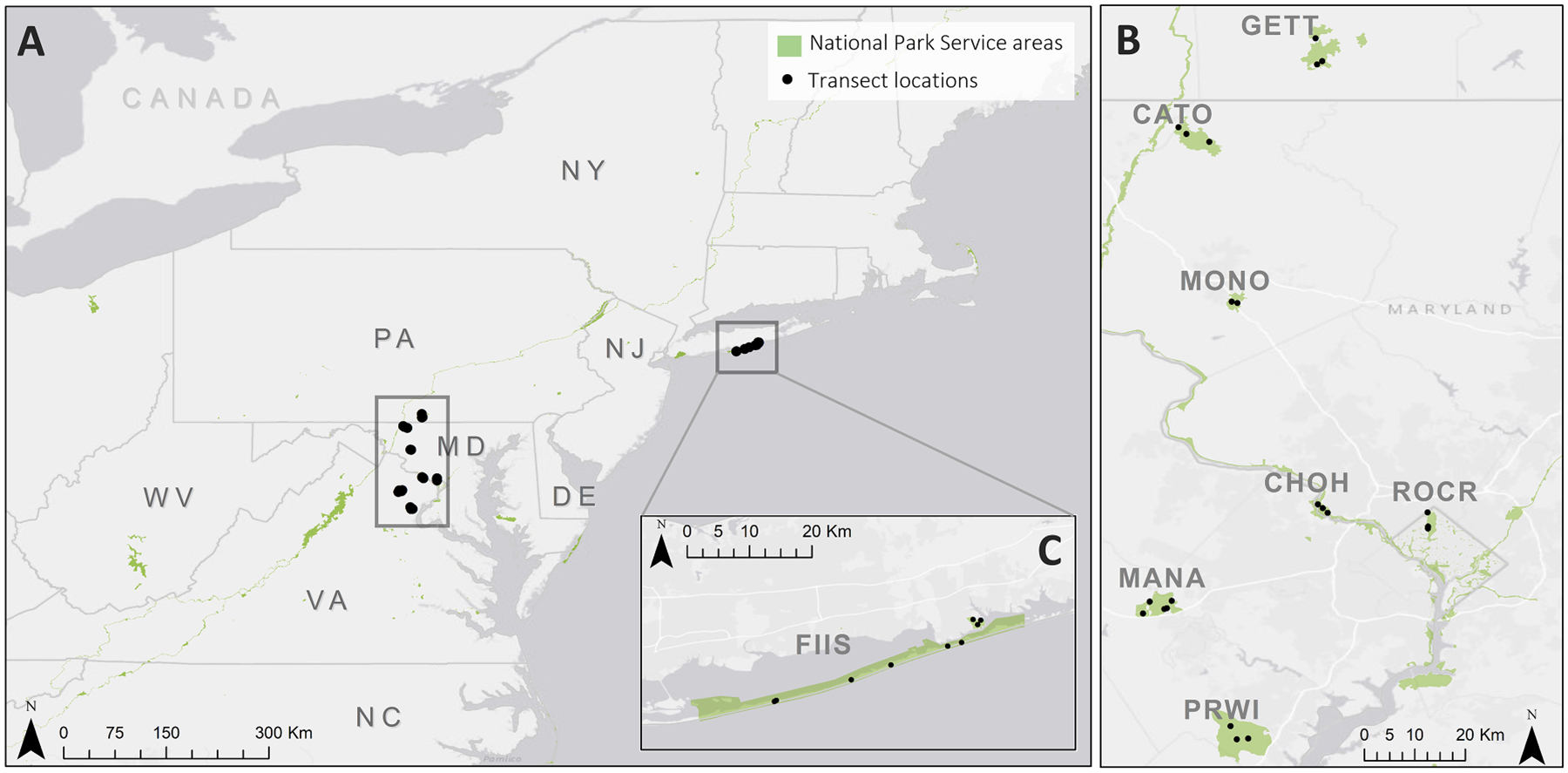
Eastern United States national park locations where annual tick surveillance occurred between 2014–2022. (**A**) Eight national parks were surveyed routinely across four states (VA, MD, PA, and NY) and one territory (District of Columbia). (**B**) Seven of the parks are located inland in the mid- and south- Atlantic regions, including Gettysburg National Military Park (GETT), Catoctin Mountain Park (CATO), Monocacy National Battlefield (MONO), Chesapeake and Ohio Canal National Historic Park (CHOH), Rock Creek National Park (ROCR), Manassas National Battlefield (MANA), and Prince William Forest Park (PRWI). (**C**, inset) Fire Island National Seashore (FIIS) is a barrier island off New York state. National Park Service boundaries are shown in green and transect locations are shown by the black points. (Basemap source: ESRI, 2022)

From 2014 to 2022, spring surveys were performed in May and June to collect host-seeking *I. scapularis* nymphs in eight national parks in the eastern United States: Catoctin Mountain Park (CATO), Chesapeake and Ohio Canal National Historic Park (CHOH), Fire Island National Seashore (FIIS), Gettysburg National Military Park (GETT), Manassas National Battlefield (MANA), Monocacy National Battlefield (MONO), Prince William Forest Park (PRWI), and Rock Creek National Park (ROCR) (Fig. 1). This research expands upon tick surveillance and pathogen prevalence reported by Johnson et al. (2017) from 2014–2015. At each park, between one and nine 750 m transects were surveyed each year using dragging techniques performed with a 1 m^2^ white drag cloth for a total area of 750 m^2^ surveyed per transect (Table 1). While vegetation features–such as ground coverage–differed visually among transects within parks, all locations were selected based on suitable tick habitat and reports of tick presence by park staff (Johnson et al., 2017), and all areas of the park were assumed to be utilized by white-tailed deer, based on anecdotal observations. Individual transect locations remained constant between years, though, not all transects were surveyed each year due to logistical limitations (Table 1; Supplementary Material I for locations). During surveys, the drag cloth was examined for ticks every 10–15 m, and all attached ticks were visually identified to life stage and species. Most transects were surveyed twice per year, once in late May and a second time approximately ten days later in an attempt to capture peak seasonal nymph activity in this region (Eisen et al., 2016; Ogden et al., 2018). Occasionally, weather prohibited either the first or second survey session from being completed. Total counts of *I. scapularis* nymphs collected were recorded per survey for each transect providing density estimates (number of ticks per 750 m^2^; Table 1). *Ixodes scapularis* ticks were collected in >90% ethanol and stored at −20°C.

All visually identified *I. scapularis* nymphs and adults were sent to the Centers for Disease Control and Prevention (CDC; Fort Collins, CO, USA) to be tested for a suite of pathogens (following Graham et al., 2016, Graham et al., 2018), including causative agents of Lyme disease (*B. burgdorferi* s.s. and *Borrelia mayonii*), relapsing fever (*Borrelia miyamotoi*), anaplasmosis (*Anaplasma phagocytophilum*), and babesiosis (*Babesia microti*). Prior to testing, all submitted *Ixodes* ticks were confirmed to be *I. scapularis* using an assay that differentiates *I. scapularis* from the morphologically similar *Ixodes affinis*, implementing a slight modification to the methods described by Wright et al. (2014). If pathogen testing results were inconclusive, the sample was not included in the analysis.

During most surveys, additional effort was made to increase nymph sample size, with the aim to acquire N=50 *I. scapularis* nymphs for *B. burgdorferi* s.s. testing per study site and year, in compliance with CDC surveillance guidelines (Centers for Disease Control and Prevention, 2021). This resulted in discrepancies between the maximum number of nymphs detected per transect and the number of nymphs submitted for pathogen testing each year (Table 1). The additional surveillance efforts were not accompanied by area surveyed, thus, density of infected nymph data–the gold standard metric used to measure acarological risk of human exposure to Lyme disease–were not available. Instead, the *I. scapularis* nymph density data and *B. burgdorferi* s.s. prevalence data were used as the response variables in four models–two models for each data type–that included environmental and deer density covariates (Table 2).

### 2.2. Environmental variables

*Ixodes scapularis* nymphal host-seeking behavior is influenced by local climatic conditions, specifically, humidity and temperature (Vail and Smith, 1998; Berger et al., 2014a, Berger et al., 2014b, Burtis et al., 2016, Eisen et al., 2016). Several weather- and climate- related metrics were considered in our models to account for environmental effects on nymph density, including proportion of hot-dry-days (pHDD), monthly relative humidity (RH), monthly Palmer’s Z-index (PZ), and monthly Palmer Drought Severity Index (PDSI). Proportion of hot-dry-days was the cumulative number of days in April and May each year when the temperature was >25°C (77°F) and no precipitation was recorded (precipitation = 0), relative to the number of days when weather data were available. Hot-dry-days were identified using precipitation and temperature data collated for each transect from the nearest National Oceanic and Atmospheric Administration (NOAA) weather station (all stations were within 10–46 km of the parks, see Supplementary Material I; NOAA, 2022b). Daily relative humidity data were acquired from the NOAA surface level humidity databases (Kalnay et al., 1996), where humidity data were recorded four times per day (at 00:00, 06:00, 12:00, and 18:00 hours) at a 2.5 degree latitude by 2.5 degree longitude grid (CHOH and PRWI fell within the same grid cell, and all other parks fell into a second grid cell). Monthly relative humidity was the sum of days each month when three of the four daily relative humidity recordings were >84% (calculated for April only, May only, and April and May; Eisen et al., 2016). This coarse relative humidity metric was used in the absence of local microclimate humidity data.

To account for long-term changes in precipitation, April and May drought indices (PDSI and PZ) were considered. PDSI is a measure of drought severity for a given month based on temperature and precipitation data and accounts for the drought risk in previous and subsequent months. A PDSI value of >4 indicates wet conditions and a value <−4 indicates drought conditions (see Heim, 2002 for calculations). PZ is a derivative of PDSI and measures the deviation of a given month from the average moisture climate for that month. PZ and PDSI were obtained from the NOAA website (NOAA, 2022c,2022d), and were estimated regionally based on the contiguous United States (CONUS) Climate Divisions (Supplementary Material I; NOAA, 2022a). All environmental variables were scaled and centered in program R v.4.1.2 using the *scale()* function (R Development Core Team, 2016).

### 2.3. White-tailed deer density

The National Capital Region (NCR) Natural Resources and Science Program engaged in annual fall monitoring to estimate white-tailed deer densities for parks in this region, including, CATO, CHOH, MANA, MONO, PRWI, and ROCR. The monitoring program started in 2000 and estimated deer densities using distance estimation procedures described in the NCR surveillance plan (NPS, 2005). Briefly, the distance sampling used driven line-transects to record observations and the distances of the observations from the sampling location. These data are used with a detection function to calculate the proportion of missed observations, which allows overall density to be estimated (Buckland et al., 2005). GETT and FIIS were not part of the NCR deer monitoring program but did estimate yearly deer density through their own natural resource program using the same distance sampling methodology. Deer density data were available at the park level for the NCR parks and GETT, and at within-park, regional level for FIIS (Supplementary Material II).

All parks but one (PRWI) engaged in active deer reduction strategies. Deer management was employed at the individual park level and began in different years for each park: 2013 for ROCR, 2016 for MONO, 2018 for CHOH and MANA, 2019 for FIIS (region WFE), and in 2020 for FIIS regions SH and WA. CATO and GETT had ongoing deer reduction management that initiated before the start of this research, and PRWI had no deer management. The number of deer removed by each park varied (Supplementary Material III). Deer density and management data were included in the models at a two-year lag, reflecting the two-year life cycle of *I. scapularis* (Fish, 1993, Wolf et al., 2020). Deer reduction efforts are expected to reduce *I. scapularis* reproductive events and therefore suppress the number of immature ticks in subsequent years, with the effects of deer management most likely to be observed for the nymphal life stage at a two-year lag. Two deer related variables were considered for the models: a two-year lagged deer density variable and a two-year lagged management variable. The two-year lagged deer density variable assigned the deer density from two years prior to a given surveillance year (e.g., if the tick surveillance occurred in 2020, the deer density from 2018 was assigned). The two-year lagged management variable was binary (0/1), with a value of 0 assigned if no deer management had occurred two or more years prior (referred to as “pre-management”), and a value of 1 assigned to surveys that occurred two or more years following the first year of deer management (referred to as “post-management”). The total number of years with deer density data included in both pre- and post- management categories can be found in Table 3. This method groups all nymph densities pre-management together and all nymph densities post-management. For some parks, post-management data extended 2-years post initial deer removals, and some extended 7-years past initial deer reduction.

### 2.4. Nymph density models

*Ixodes scapularis* nymph count data were associated with 750 m^2^ surveillance areas, thus we use “density” to describe these data, though raw counts were used in these models. Nymph densities were used in two question-focused models designed to address (i) the effect of deer density on *I. scapularis* nymph densities and (ii) the impact of deer reduction management on nymph densities. For both models, the response variable was the maximum nymph density observed at each transect among survey sessions within a given year. Maximum nymph density was the maximum number of nymphs observed at each 750 m^2^ transect across all sampling sessions (i.e., maximum nymph density could be obtained from different sampling sessions for transects within a park), and all transects were included as replicates within parks. Maximum density was used to minimize variability introduced by sampling before, or after, peak nymph emergence. By using maximum density, we aimed to understand the highest potential acarological risk of encountering a nymph, using nymph density as a proxy. Model selection methods were not implemented because the models were hypothesis driven. To determine informative environmental variables, preliminary models were executed whereby maximum nymph density (nymph count per 750 m^2^) was the response variable, and only environmental variables were included as predictor variables (excluding all other variables). This approach was implemented to account for the influence of climate on nymph densities without overfitting the final model. Environmental variables were retained in the final model if their 95% credible interval did not overlap with zero in the preliminary model. Final environmental covariates were not highly correlated (correlation coefficient, r≤0.75).

To understand if variation in deer density affected the density of host-seeking *I. scapularis* nymphs, a Bayesian generalized linear model with group-specific terms was utilized (model 01). Nymph density data for transect survey *i* in park *j*, were assumed to follow a negative binomial distribution with a mean μ_*ij*_ and an overdispersion parameter φ (≥0). Negative binomial regressions account for over-dispersed data, which is often observed with count datasets. The log link function was implemented to relate the response variable to the linear combination of the predictors: the intercept (β_0_), corresponding to the logged mean number of nymphs observed; the effect of the proportion of hot-dry-days (pHDD_*ii*_) on nymph count (β_1_); the effect of April relative humidity (RH_April_*ii*_) on mean nymph count (β_2_), and the effect (β_3_) of park specific lagged deer density (deer_dens_*ij*_) on mean tick density (see Table 2). To account for park level effect and multiple transect locations within parks, park identity was included as a random effect (intercept), ρ_*j*_. Model 01 was designed to understand the general trend of deer density impact on nymph density, not the effect at the park level, thus park was included as a random effect. The final model structure was as follows:

$${\text{density}}_{ij}\sim NB\left(\mu_{ij},\phi \right)$$

(1) $$\text{log}\left(\mu_{ij}\right)=\beta_{0}+\beta_{1}\times pHDD_{ij}+\beta_{2}\times RH\_{\text{April}}_{ij}+\beta_{3}\times \text{deer}\_{\text{dens}}_{ij}\text{+}\rho_{j}$$

A second Bayesian generalized linear model was used to address the impact of park-specific deer management on tick density (model 02). In this model, only parks with at least two years of nymph density data following the initiation of deer reduction efforts (post-management) were included. These parks included CHOH, MANA, MONO, FIIS (region WFE), and ROCR (Table 3; see Supplementary Material III for deer removal effort). Similar to the first model, nymph density data for transect survey *i* were assumed to follow a negative binomial distribution with a mean M_*ij*_ and an overdispersion parameter Φ (≥0). The log link function was implemented to relate nymph densities to the linear combination of the fixed effects: the intercept (B_0_), corresponded to the logged mean density of nymphs observed at the reference park; the effect of the proportion of hot-dry-days (pHDD_*ij*_) on nymph density (B_1_); the effect of April relative humidity (RH_April_*ij*_) on mean nymph density (B_2_), and the effect (B_3_) of the interaction between park identity (Park_ID_*j*_) and deer management (binary term; deer_manage_*ij*_; 0, no management; 1, management two or more years prior) (Table 2). Model 02 differs from model 01 in that the effect of deer removal on nymph density at each park was of interest, so park was included as an independent variable (interaction) instead of a random effect. The final model structure was as follows:

$${\text{density}}_{ij}\sim NB\left(M_{ij},\Phi \right)$$

(2) $$\text{log}\left(M_{ij}\right)={\text{B}}_{0}+{\text{B}}_{1}\times pHDD_{ij}+{\text{B}}_{2}\times RH\_{\text{April}}_{ij}+{\text{B}}_{3}\times \text{deer}\_{\text{manage}}_{ij}\times \text{Park}\_ID_{j}$$

### 2.5. Pathogen models

Using the *B. burgdorferi* s.s. pathogen data, two models were designed to understand (i) the effect of deer density on infection prevalence and (ii) the effect of deer reduction strategies on infection prevalence. Similar to the density models, environmental variables were assessed in a preliminary model and only significant variables were included in the final pathogen model. Pathogen data were only included in the model if the number of ticks submitted for testing was N>1 for a given year (Table 1). This sample size is lower than CDC recommendation (N=50), which is intended for making conclusions about a specific prevalence estimate in a given year or locale. Our interest, however, is in making inferences across multiple years and locations for the effect of deer reductions, and removing park-years below certain sample size thresholds may bias conclusions if there is a correlation between infection prevalence and the number of ticks collected. The N>1 sample size threshold produced the same trends as a more stringent sample size (N≥10; Supplementary Materials IV) and allowed for the retention of surveillance sites that experienced declines in nymph densities following management (e.g., MANA and MONO). In the first model, the pathogen data were modeled using a Bayesian generalized linear model with group-specific terms (model 03). The response variable was a vector of successes to failures (*B. burgdorferi* s.s. positive versus negative nymphs) during survey *i* at park *j* (Bbss_pos_*ij*_|Bbss_neg_*ij*_), following a binomial distribution with a mean probability of success, ω_*ij*_, given the number of attempts (ψ; total number of nymphs tested; sum of Bbss_pos_*ij*_ and Bbss_neg_*ij*_). The model’s fixed effects included an intercept (η_0_), corresponding to the average number of *B. burgdorferi* s.s. positive nymphs; the effect of May moisture anomalies (PZ_May_*ii*_; η_1_), April relative humidity (RH_April_*ij*_; η_2_), and May relative humidity (RH_May_*ij*_; η_3_) on the prevalence of *B. burgdorferi* s.s.; and the effect of lagged deer density (deer_dens_*ij*_; η_4_). Park identity was included as a random effect (P_*j*_), as the general effect of deer density–not park specific effects–on pathogen prevalence was of interest. The logit function was implemented to relate the linear combination of the predictors to the response variable (Table 2). The final model structure was:

$$\text{Bbss}\_pos_{ij}|\text{Bbss}\_neg_{ij}\sim BIN\left(\omega_{ij},\psi \right)$$

(3) $$\text{logit}\left(\omega_{ij}\right)=\eta_{0}+\eta_{1}\times PZ\_May_{ij}+\eta_{2}\times RH\_{\text{April}}_{ij}+\eta_{3}\times RH\_May_{ij}+\eta_{4}\times \text{deer}\_dens_{ij}\text{+}{\text{P}}_{j}$$

To address the impact of deer removal on prevalence of *B. burgdorferi* s.s. in nymphs, a second pathogen Bayesian generalized linear model was implemented including only parks with at least two years of pathogen data post-management (model 04). These parks included CHOH, MANA, MONO, FIIS (region WFE), and ROCR (Table 3). The response variable was a vector of successes to failures (*B. burgdorferi* s.s. positive nymphs, Bbss_pos_*ij*_*; B. burgdorferi* s.s. negative nymphs, Bbss_neg_*ij*_) following a binomial distribution with a mean probability of success, Ω_*ij*_, given the number of attempts (Ψ; total number of nymphs tested; sum of Bbss_pos_*ij*_ and Bbss_neg_*ij*_). The model’s fixed effects included an intercept (H_0_), corresponding to the average number of *B. burgdorferi* s.s. positive nymphs at the reference park; the effect of May moisture anomalies (PZ_May_*ij*_; H_1_), April relative humidity (RH_April_*ij*_; H_2_), and May relative humidity (RH_May_*ij*_; H_3_); and the interaction between park identity (Park_ID_*j*_) and the binary lagged deer management term (deer_manage_*ij*_; H_4_). The inclusion of an interaction between park identity and deer management allowed for the assessment of within-park deer-removal effort on nymph densities. This model had no random effect terms (Table 2). The final model structure was as follows:

$$\text{Bbss}\_pos_{ij}|\text{Bbss}\_neg_{ij}\sim BIN\left(\Omega_{ij},\Psi \right)$$

(4) $$\text{logit}\left(\Omega_{ij}\right)={\text{H}}_{0}+{\text{H}}_{1}\times PZ\_May_{ij}{\text{+H}}_{\text{2}}\times RH\_{\text{April}}_{ij}{\text{+H}}_{\text{3}}\times RH\_May_{ij}{\text{+H}}_{\text{4}}\times \text{deer}\_{\text{manage}}_{ij}\times \text{Park}\_ID_{j}$$

### 2.6. Model execution and predictions

For all models, priors were weakly informative for fixed variables: N (0, 2.0; mean and standard deviation). The priors on the covariance matrices of the group-specific terms (random effects) were uninformative, with regularization, concentration, scale, and shape set to 1. Models were fit in R v.4.1.2 using function *stan_glmer()* in package rstanarm v.2.21.3 (R Development Core Team, 2016, Brilleman et al., 2018, Goodrich et al., 2022) and were run for 30,000 Markov chain Monte Carlo iterations with three chains, following a 30,000-iteration warmup (burn-in). Model convergence was confirmed for all models through visual inspection of parameter trace plots and ensuring the Gelman and Rubin’s potential scale reduction factor ($\hat{R}$) values were near to 1 (<1.1 Brooks and Gelman, 1998). Further, all variables had an effective sample size of ≥20,000. Variables were considered important (or, “significant”) predictors of the dependent variable if their posterior credible intervals did not overlap with zero. Post-hoc analyses were conducted to understand the effect of the interaction between the binary deer management variable and park identity. Estimated marginal means (Least-Squares means) were calculated using the *emmeans()* function in R package emmeans v.1.7.3 (Lenth, 2022).

For all predictions, the *posterior_predict()* function in R package rstanarm v.2.21.3 was implemented (Goodrich et al., 2022). Mean *I. scapularis* nymph density (number of ticks per 750 m^2^) was predicted using model 01 for each park, addressing the effect of white-tailed deer density at each park. Nymph densities were predicted across a range of deer densities, from 5 through 85 deer per km^2^ at intervals of 5 deer per km^2^, and yearly for each park using the corresponding lagged deer density for 2014–2022. Environmental covariates were held constant at their means for all predictions. These predictions were used to understand the effect of deer density in reducing tick density, specifically focusing on deer densities of 5 and 20 deer per km^2^ (commonly cited deer density thresholds). Similarly, using model 03, the mean prevalence of *B. burgdorferi* s.s. infection in *I. scapularis* nymphs was predicted across the same range of deer density values for each park (5–85 deer per km^2^ at intervals of five) and yearly at all parks.

To understand the impact of deer management on tick densities and *B. burgdorferi* s.s. prevalence, mean peak *I. scapularis* nymph densities (per 750 m^2^) were predicted using model 02 under scenarios of no white-tailed deer management 2-years prior and deer removal 2-years prior (values 0 and 1, respectively, also referred to as “pre-management“ and “post-management”) for each park. Similarly, using model 04, the mean number of *I. scapularis* nymphs infected with *B. burgdorferi* s.s. was predicted for both management scenarios (0 and 1) with the total number of nymphs (number of attempts) held constant (N=100).

### 2.7. Density of infected nymphs (DIN)

The density of infected nymphs (DIN; per 750 m^2^) was calculated by combining the prediction estimates from tick density and pathogen prevalence models (models 01 and 03). The DIN pre- and post- white-tailed deer management was calculated by combining predictions of nymph density and *B. burgdorferi* s.s. prevalence from models 02 and 04. For computational purposes, values from prediction posteriors were subset to 1,000 (from 90,000).

## 3. Results

### 3.1. Tick surveillance and deer density

**Fig. 2.**
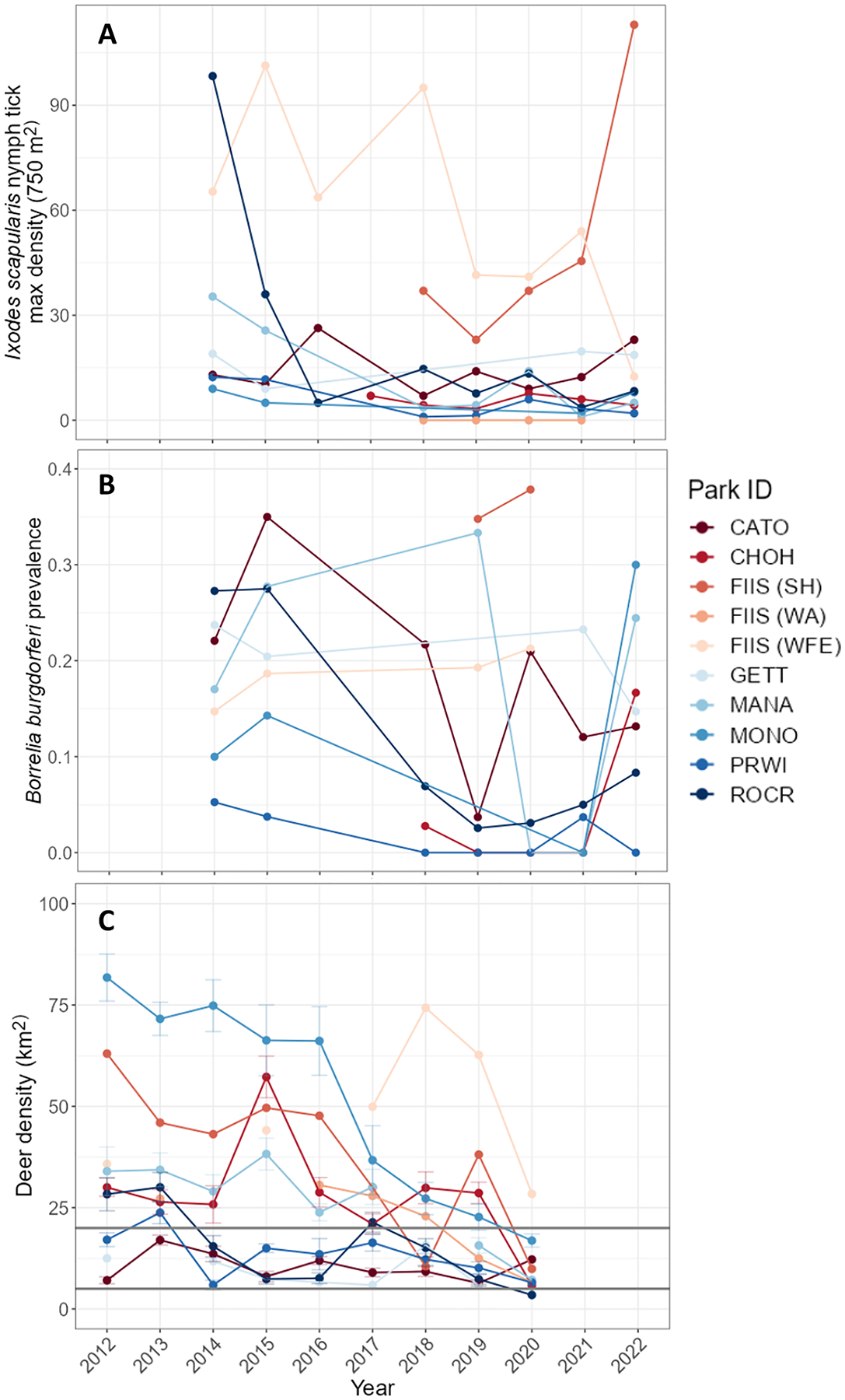
Densities of *Ixodes scapularis* nymphs (per 750 m^2^), *Borrelia burgdorferi* sensu stricto infection prevalence, and white-tailed deer densities (per km^2^) at eight United States national parks from 2012–2022. (**A**) Tick density data represent the average of the maximum densities detected at each transect within a given park or park region (transect number surveyed ranged from 1 to 9) per year and were available from 2014–2022. Mean tick densities are plotted as points (see Supplemental Material X for mean log[tick density] and S.D.). (**B**) Average *B. burgdorferi* s.s. infection prevalence among *I. scapularis* nymphs per park from 2014–2022. (**C**) White-tailed deer density estimates (±S.E.) were provided by the National Capital Region Wildlife Resources Program for each national park from 2012–2020. Horizontal gray lines are placed at deer density values of 5 and 20 per km^2^. See Table 1 for full park names.

**Fig. 3.**
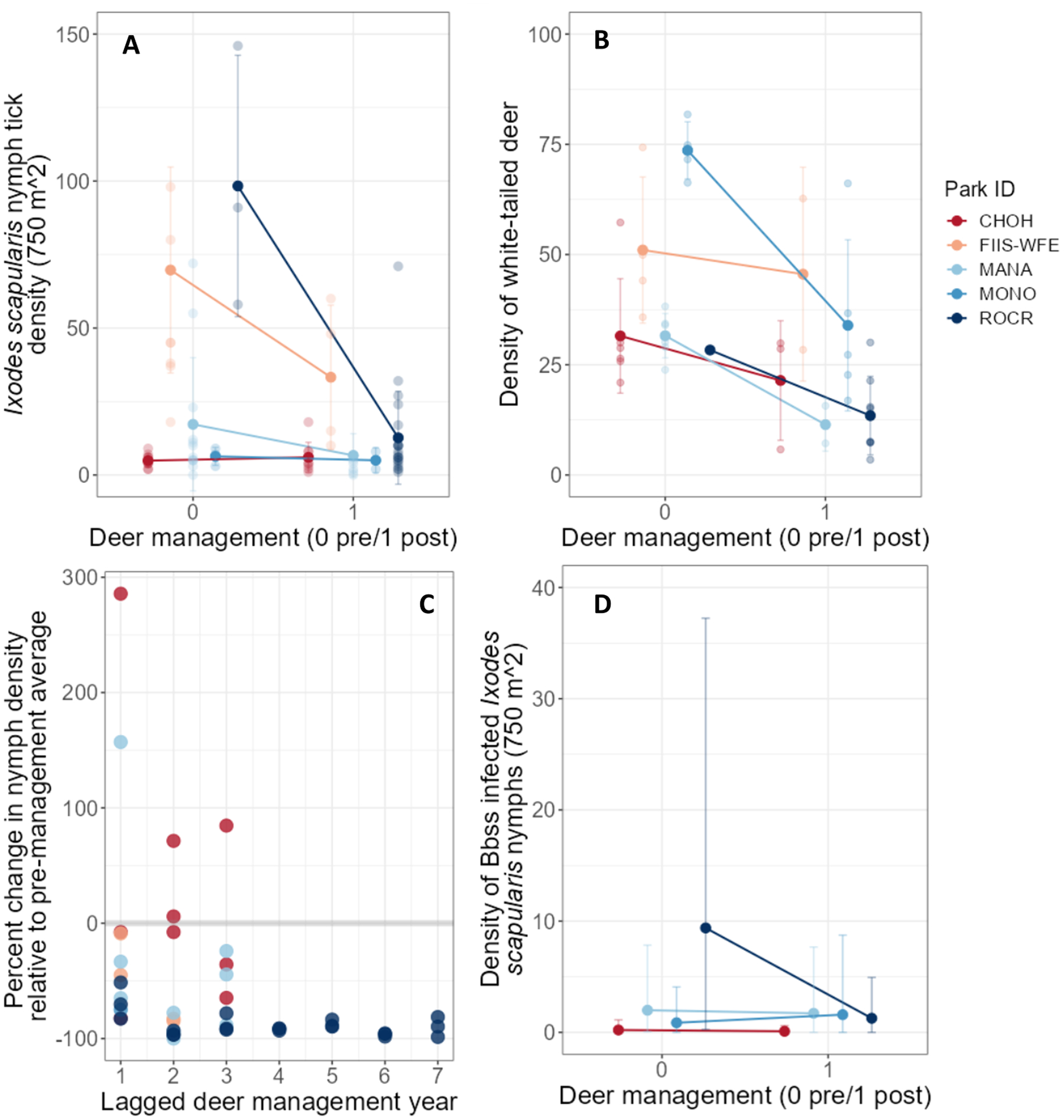
The effect of white-tailed deer reduction efforts on *Ixodes scapularis* nymph density and *Borrelia burgdorferi* sensu stricto infection prevalence from 2014–2022. (**A**) The average density of nymphs (per 750 m^2^; ±S.D.) from field observations pre- and post- deer management. Individual observations are shown by the semi-transparent points. (**B**) The average white-tailed deer density (±S.D.) pre- and post- management efforts for each park or park region. (**C**) Percent change in nymph density between each post-management year and the average nymph density observed pre-management. Each data point represents percent changes for a single transect within each park relative to the average nymph density for that respective transect pre-management. The x-axis represents the 2-year lagged number of years following the first year of management (e.g., if management started in 2013, x-axis value of 1 indicates change in nymph densities observed in 2015). (**D**) Predicted density of infected nymphs (per 750 m^2^; with 95% credible interval) pre- and post- deer management efforts.

Between 2014 and 2022, a total of 30 transects were surveyed for *I. scapularis* nymphs with 164 total data points (Martin et al., 2023). Transect locations were consistent across years though the number of transects surveyed varied by park (Table 1). The maximum density of *I. scapularis* nymphs varied across parks and transects (Fig. 2, A; Table 1), with the range of maximum nymph densities per transect between 0–153 per 750 m^2^, and the average nymph density at 16 ticks per 750 m^2^ (7 median, 15.9 mean, 24.3 SD). Nymph densities also varied pre- and post- deer management (Fig. 3, A, C). Pathogen data were not collected every year for each park and transect (total pathogen prevalence data points N=118; Tables 1 and 3), and after filtering by sample size (N>1 nymph submitted), 112 *B. burgdorferi* s.s. prevalence data points remained (sample thresholds of N>1 and N≥10 yield the same trends; Supplementary Material IV). Prevalence of *B. burgdorferi* s.s. in *I. scapularis* nymphs ranged from 0 to 50% across parks (9.2% median, 13.3% mean, 13.4% S.D.; Fig. 2, B; Table 1).

White-tailed deer densities varied across national parks and park regions through time (Fig. 2, C), and were available at the park level for all parks and at within-park regional levels for FIIS. The range (minimum and maximum) of deer densities observed for each park (deer per km^2^) from 2012–2020 were as follows: CATO 6–17, CHOH 6–57, FIIS 7–74 (across regions), GETT 6–16, MANA 7–38, MONO 17–82, PRWI 6–24, and ROCR 3–30 (Fig. 2, C). The average deer density across all parks and park regions between 2012 and 2020 was 26 deer per km^2^ (26.0 mean, 19.4 S.D.). During this period (2012–2020), only one park had a deer density of <5 deer per km^2^ (ROCR), and it was for a single year (2020); however, most parks and park regions hosted deer densities of <20 deer per km^2^, through management efforts or by natural population fluctuations (Fig. 2, C). All but one park engaged in deer removal practices, and the number of deer removed varied among parks and years (Supplementary Material III). Deer reduction efforts varied across these parks or park regions between 2012–2020, with the number of years when removal occurred ranging from 2 to 8, and the total number of deer removed ranging from 158–659 during this period (Table 3; Supplementary Material III). All parks that engaged in deer reduction experienced a decline in deer densities following management implementation (Fig. 3, B).

### 3.2. Deer density and nymph density

**Fig. 4.**
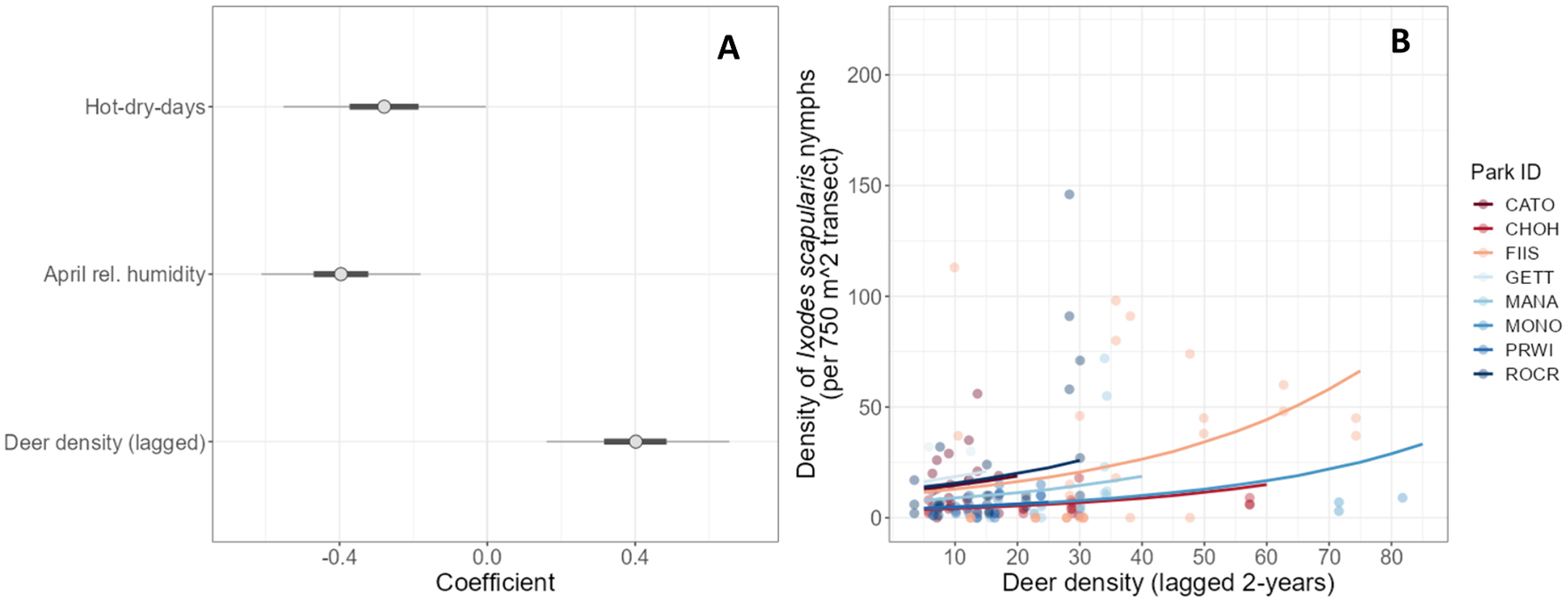
Model results and predictions assessing the effect of deer density on *Ixodes scapularis* nymph densities. (**A**) Coefficient plot for model 01, investigating the impact of 2-year lagged deer density on nymph densities. The plot shows the mean point estimate, the 50% probability mass interval (thick, gray line), and the 95% probability mass interval (thin, grey line). (**B**) Nymph density predictions across a range of deer densities (5–85 per km^2^) using model 01 results, extrapolated to the maximum deer density observed for each park. Mean predicted values are presented by the solid lines and raw data are shown by the points (both predicted means and raw data colored by park). See Table 1 for full park names.

The effect of deer density on *I. scapularis* host-seeking nymph density was assessed using model 01. This model revealed that lagged deer density was significantly positively associated with nymph density (β_3_ = 0.40 [95% posterior credible interval (PCI) = 0.16, 0.66]), resulting in a 49% increase ([(exponentiated coefficient – 1) × 100]; UCLA Statistical Consulting Group) in the number of *I. scapularis* nymphs per 750 m^2^ with a 1 SD increase in 2-year lagged deer density (16.2 deer per km^2^ S.D., for data points included in model). Both the proportion of hot-dry-days and April relative humidity had significant negative effects on nymph counts (β_1_ = −0.28 [95% PCI = −0.55, −0.00]; β_2_ = −0.40 [95% PCI = −0.61, −0.18]), with 24% and 33% reduction in nymphs with a 1-SD increase in each, respectively (Fig. 4, A). The intercepts associated with the random effect of park ID ranged from −0.71 to 0.70, with GETT and ROCR having the largest positive intercepts (i.e., these parks had higher nymph densities than the average park) and MONO and CHOH having the largest negative intercepts (i.e., these parks had lower nymph densities than the average park; Supplementary Material V).

Predictions of tick densities at deer densities from 5 to 85 deer per km^2^ varied across parks (Fig. 4, B). At a density of 5 deer per km^2^, *I. scapularis* nymph densities ranged between 4–16 nymphs per 750 m^2^ across all parks, with the highest densities observed at GETT and ROCR (${\bar{x}}_{\text{GETT}}=16$ [95% PCI = 0, 76]; ${\bar{x}}_{\text{ROCR}}=14$ [95% PCI = 0, 62]) and lowest at MONO and CHOH (${\bar{x}}_{\text{MONO}}=4$ [95% PCI = 0, 23]; ${\bar{x}}_{\text{CHOH}}=4$ [95% PCI = 0, 18]). At a deer density of 20 deer per km^2^, densities of 5–24 nymph *I. scapularis* ticks per 750 m^2^ were observed across parks, with highest predicted densities of ticks observed at GETT and ROCR (${\bar{x}}_{\text{GETT}}=24$ [95% PCI = 0, 109]; ${\bar{x}}_{\text{ROCR}}=20$ [95% PCI = 0, 87]) and the lowest nymph densities observed at MONO and CHOH (${\bar{x}}_{\text{MONO}}=6$ [95% PCI = 0, 31]; ${\bar{x}}_{\text{CHOH}}=5$ [95% PCI = 0, 25]).

### 3.3. Deer management and nymph density

To understand the binary effect of deer reduction practices on nymph densities, a second model was implemented (model 02). Four of five parks experienced a reduction in nymphs following deer removal. Post-hoc analysis revealed the contrast in estimated marginal mean peak tick densities when no deer management occurred (pre), and following deer reduction (post), was only significant for one park, ROCR (${\bar{x}}_{\text{ROCR\_pre-post}}=1.37$ [95% PCI =0.52, 2.23]). Contrasts in estimated marginal mean tick densities for the other four parks and park regions (MONO, CHOH, MANA, and FIIS [WFE]) were not significant (Supplementary Material VI). For ROCR, when no deer management was implemented, the predicted mean value of nymphs was 58 (95% PCI = 3, 206), which decreased to 14 nymphs following deer management implementation (95% PCI = 0, 47; Table 3). For MONO, MANA, and FIIS (WFE), there was a general trend for a decrease in the density of ticks following management (${\bar{x}}_{\text{MONO\_pre}}=9$ [95% PCI = 0, 37], ${\bar{x}}_{\text{MONO\_post}}=6$ [95% PCI = 0, 29]; ${\bar{x}}_{\text{MANA\_pre}}=16$ [95% PCI = 0, 55], ${\bar{x}}_{\text{MANA\_post}}=9$ [95% PCI = 0, 34]; ${\bar{x}}_{\text{FIIS-WFE\_pre}}=76$ [95% PCI = 4, 256], ${\bar{x}}_{\text{FIIS-WFE\_post}}=48$ [95% PCI = 2, 180]). For CHOH, the density of nymphs increased slightly following management (Table 3). Neither of the climate variables–proportion of hot-dry-days nor April relative humidity–had significant effects on the density of nymphs (Supplementary Material VI; B_1_ = 0.05 [95% PCI = −0.25, 0.37]; B_2_ = −0.16 [95% PCI = −0.41, 0.09]).

### 3.4. Deer density and B. burgdorferi s.s. prevalence

The effect of deer density on *B. burgdorferi* s.s. prevalence in questing *I. scapularis* nymphs was assessed using model 03. Lagged deer density did not significantly affect the *B. burgdorferi* s.s. prevalence in *I. scapularis* nymphs (Supplementary Material IV; η_4_ = 0.04 [95% PCI = −0.15, 0.23], odds ratio 1.04). April relative humidity was significantly negatively associated with *B. burgdorferi* s.s. prevalence (η_2_ = −0.14 [95% PCI = −0.28, −0.01]) with an odds ratio of 0.87 (ratio of 1 indicating no change, >1 indicating increase, and <1 indicating decrease), but May moisture anomaly and May relative humidity were not (η_1_ = −0.16 [95% PCI = −0.39, 0.07]; η_3_ = −0.00 [95% PCI = −0.25, 0.23], respectively). The intercepts for the random effect of park ranged from −1.47 to 0.60, with GETT and FIIS having the largest positive intercepts (i.e., a higher proportion of *B. burgdorferi* s.s. positive nymphs than the average park) and PRWI and CHOH having the largest negative intercepts (i.e., a lower proportion of *B. burgdorferi* s.s. positive nymphs than the average park; Supplementary Material IV). The predicted prevalence of *B. burgdorferi* s.s. infected *I. scapularis* nymphs increased slightly–not significantly–with deer density when environmental variables were held constant (Supplementary Material IV).

### 3.5. Deer management and B. burgdorferi s.s. prevalence

To understand the effect of park specific deer reduction efforts on *B. burgdorferi* s.s. infection prevalence in *I. scapularis* nymphs, a second pathogen model was implemented (model 04). Post-hoc analysis of marginal means revealed that deer management did not result in significant reductions in *B. burgdorferi* s.s. prevalence between pre- and post- management in any park, but ROCR and CHOH did experience general declines in *B. burgdorferi* s.s. prevalence following deer reduction efforts. When no deer management was implemented, the predicted prevalence of *B. burgdorferi* s.s. at ROCR and CHOH were 17 and 4%, respectively, which was reduced to 9 and 1% following deer management. MONO and MANA experienced a general increase in *B. burgdorferi* s.s. prevalence following deer reductions, with MONO increasing from 10 to 22% and MANA increasing from 12 to 17%, each with wide credible intervals (Supplementary Material VI). April relative humidity significantly reduced the prevalence of *B. burgdorferi* s.s. in nymphs (Supplementary Material VI; H_2_ = −0.70 [95% PCI = −1.19, −0.31]), with an odds ratio of 0.50 with a 1-SD unit increase in April relative humidity. May moisture anomaly and May relative humidity did not affect *B. burgdorferi* s.s. prevalence (H_1_ = −0.42 [95% PCI = −0.93, 0.07]; H_3_ = 0.22 [95% PCI = −0.32, 0.75]).

### 3.6. Density of infected nymphs (DIN) and white-tailed deer

**Fig. 5.**
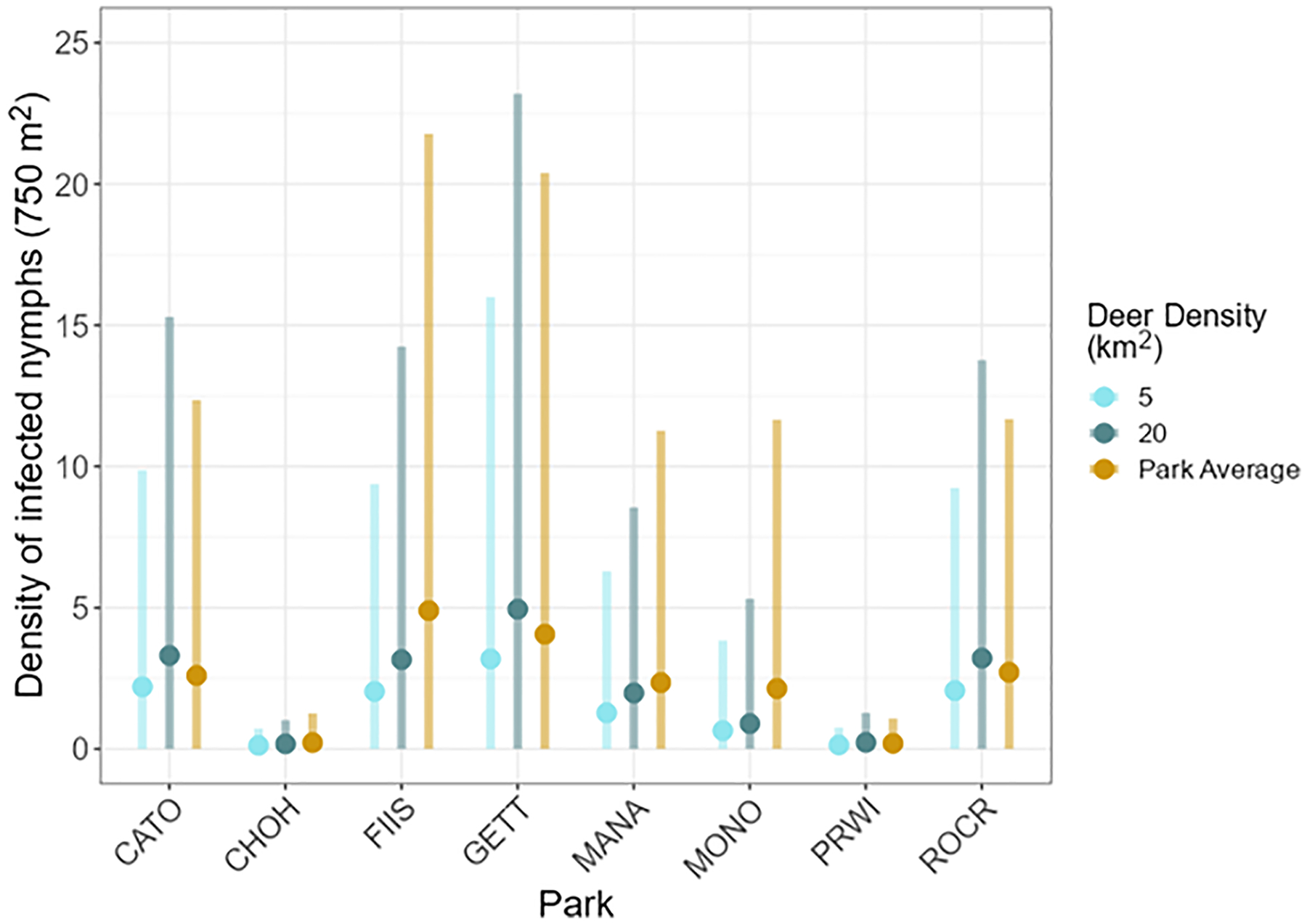
The density of *Borrelia burgdorferi* sensu stricto-infected *Ixodes scapularis* nymphs (DIN) at varying deer densities for eight United States national parks. DIN was estimated for each park at deer densities of 5 (light blue) and 20 (dark blue) deer per km^2^, and at the average deer density at each park from 2012–2020 (gold). The mean predicted densities are shown by points and the credible intervals are shown by the vertical semi-transparetn bars. The average deer density for each park was: CATO 10.50, CHOH 28.17, FIIS 37.55 (region SH), GETT 9.40, MANA 26.56, MONO 51.58, PRWI 13.40, and ROCR 15.13. See Table 1 for full park names.

DIN was predicted for all parks at deer densities of 5 and 20 deer per km^2^, and for each park at that park’s mean deer density from 2012–2020 (Fig. 5) using prediction results from models 01 and 03. At 5 deer per km^2^, the mean DIN at all parks was 3 or fewer per 750 m^2^. The highest mean DIN was at GETT, with 3.18 infected nymphs per 750 m^2^, and the lowest at CHOH (0.12 infected nymphs per 750 m^2^). Similarly, at 20 deer per km^2^, no park exceeded a mean DIN of 5 infected nymphs per 750 m^2^. The highest mean DIN at 20 deer per km^2^ was observed at GETT and lowest at CHOH (4.95 and 0.18 nymphs per 750 m^2^, respectively). The predicted DIN per 750 m^2^ pre- and post- deer management was estimated using output from models 02 and 04. Following deer management, the DIN varied among parks (Fig. 3, D; Table 3).

## 4. Discussion

The efficacy of managing white-tailed deer density as a strategy for mitigating Lyme disease risk in humans is complicated (Kugeler et al., 2015). Here, we used nine years of *I. scapularis* nymph density and *B. burgdorferi* s.s. infection prevalence data from eight national parks and park regions to better understand the effect of white-tailed deer density and reduction efforts on the density of infected nymphs in the landscape. Our results show that 2-year lagged deer density was significantly, positively correlated with nymph densities, with a 49% increase in nymph densities observed with a 1 S.D. increase in deer density (on average, approximately 16 deer per km^2^); the largest effect among considered predictor variables. Conversely, deer density was not significantly correlated with *B. burgdorferi* s.s. infection prevalence across parks. Further, we found that the DIN remained at ≤5 *B. burgdorferi* s.s.-infected nymphs per 750 m^2^ for all parks at white-tailed deer densities of 20 deer per km^2^ or less; a deer density observed at almost all parks. At the park level, deer management efforts resulted in significant reduction in nymph densities in one park with general trends of decline in three others but had variable effects on *B. burgdorferi* s.s. prevalence. Our findings suggest that white-tailed deer density and management can play an important role in controlling *I. scapularis* nymph density, but additional factors may be important in decreasing *B. burgdorferi* s.s. prevalence.

### 4.1. White-tailed deer density significantly influences I. scapularis nymph densities across parks

There has been much debate about the utility in managing white-tailed deer densities as a method to reduce nymph densities and Lyme disease risk. In the absence of complete elimination of white-tailed deer, the effect of deer management has been mixed (see review by Kugeler et al., 2016). Several foundational studies have demonstrated a positive relationship between deer densities and nymph densities at limited scales (e.g., island population; Wilson et al., 1984, Wilson et al., 1988). Here, we demonstrate a significant, positive association between 2-year lagged white-tailed deer densities and *I. scapularis* nymph densities across a broad (>300 km) geographic scale at eight national parks or park regions in the eastern United States.

While all parks experienced increasing nymph densities with increasing deer densities, there was substantial variation in tick densities across parks, with the highest densities observed at GETT, ROCR, and CATO, and the lowest densities observed at PRWI, CHOH, and MONO. These variations are likely due to local environmental differences among parks that are not explored here, given the focus of this study. Briefly, variation in vegetation type (Ginsberg et al., 2020) and the amount of forest fragmentation (Ferrell and Brinkerhoff, 2018), among other factors, could have influenced the variation in density of *I. scapularis* nymphs observed across parks. Further, there was variation within parks among survey transects within years. While all parks experienced annual fluctuations in nymph densities, the general trend was decreasing nymph densities throughout this study period, at least partly mirroring declining deer densities across parks.

### 4.2. White-tailed deer removal likely reduces nymph densities

White-tailed deer reduction efforts resulted in declines in tick densities at four of the five national parks or park regions that engaged in deer management; a significant reduction in tick density was observed in one of five parks (ROCR), a non-significant reduction was observed in two parks (FIIS and MANA), and no appreciable change was detected in the remaining two parks (CHOH and MONO). While only ROCR experienced a significant reduction in nymph densities following white-tailed deer removal, MONO, MANA, and FIIS (region WFE) all experienced general declines in nymph densities following management. ROCR engaged in eight consecutive years of deer removal and removed 505 deer in total, reducing deer densities from 28.3 deer per km^2^ in 2012 to 3.47 deer per km^2^ in 2020. MONO, MANA, and FIIS (WFE) removed 659, 451, and 259 deer over five, three, and two years, respectively. During their management periods, MONO decreased deer densities from 66.14 deer per km^2^ in 2016 to 16.89 deer per km^2^ in 2020; MANA reduced deer densities to 7.17 deer per km^2^ in 2020 (first year of management density data are missing); and in one-year of management, FIIS region WFE reduced deer densities from 62.70 deer per km^2^ in 2019 to 28.40 deer per km^2^ in 2020. CHOH was the only park to experience a slight, non-significant, increase in nymph densities following three years of management and removal of 158 deer (deer densities reduced from 29.89 deer per km^2^ in 2018 to 5.79 deer per km^2^ in 2020). However, pre-management, CHOH had a low density of ticks (5 nymphs per 750 m^2^), which only increased by one nymph per 750 m^2^ post-management (6 nymphs per 750 m^2^); a shift that could be attributed to natural, inter-annual fluctuations in tick populations (Deblinger et al., 1993).

Notably, there was no standardized deer reduction protocol implemented across parks, and substantial variability was observed in white-tailed deer removal efforts among parks and across years within parks. This study represents a real-world implementation of deer management for nymph control and revealed a general trend of reduced nymph densities following deer removal. The variability in the magnitude of this effect across parks may in part be explained by a potential delay following management implementation. Foundational studies reported gradual declines in nymph densities following deer reductions (Wilson et al., 1988, Deblinger et al., 1993), generally observable two or more years following management (3–4 years for significant reductions). While these studies used nymph counts from small mammal hosts as the metric of abundance–which is not predictive of questing nymphs (Ginsberg et al., 2020)–similar trends could be occurring in questing nymphs, and further declines may be observed in coming years for parks that recently initiated deer management (3 or fewer years). Specifically, successive years with removal of deer may result in successive reduction in nymphs, and the true magnitude of this effect may not be observed in the first few years following management and may be dampened by combining all densities post-management into one group. Our results follow this trend, with larger percent changes in nymph densities observed in years 2–3 post-management relative to the first year; however, the interpretation is limited due to three of the five parks having only three years of data post-management.

The effect of white-tailed deer management on nymph densities may be dampened in areas where environmental factors limit nymph abundance. Notably, MONO and CHOH supported very few nymphs prior to management efforts (9 and 5 per 750 m^2^, respectively). Following deer management, MONO experienced the smallest percent decrease in nymph density among parks, and CHOH experienced a slight increase in nymph density (6 nymphs per 750 m^2^ for each park). This suggests that lowering the density of the reproductive host (white-tailed deer) may not be a significant factor in controlling nymph densities if densities are already low or constrained due to other abiotic or biotic factors. Other factors known to influence nymph densities include vegetation and woody debris (invasive plants, Allan et al., 2010;; coarse woody debris, Larson et al., 2022), leaf litter (Schulze et al., 1995), small mammal hosts (Ostfeld et al., 2006), and forest characteristics (canopy cover, Ginsberg et al., 2020); factors that likely vary across our study range and contribute to the variable tick densities observed across parks.

### 4.3. B. burgdorferi s.s. prevalence: influenced by more than white-tailed deer

Lagged white-tailed deer density did not have a significant effect on *B. burgdorferi* s.s. infection prevalence in this study, nor did white-tailed deer reductions change the prevalence of *B. burgdorferi* s.s. in parks that engaged in management. However, there was a weak positive trend of increased *B. burgdorferi* s.s. prevalence with increasing deer density. Currently, there is a paucity of studies examining the relationship between deer density and *B. burgdorferi* s.s. infection rates in *I. scapularis*. Literature from a similar system–*I. ricinus* and *B. burgdorferi* s.l. in Europe–have reported mixed results, where some studies reported no relationship between deer density and pathogen prevalence in ticks (Pichon et al., 1999), some found a positive relationship between deer density and *B. burgdorferi* s.l. (James et al., 2013), and some found a negative relationship (lower *Borrelia* prevalence in areas with higher deer densities; Rosef et al., 2009, Mysterud et al., 2013); though, deer density ranges among these studies likely differed. Further, we report discrepancies in *B. burgdorferi* s.s. among parks engaging in white-tailed deer reduction efforts. Specifically, two parks experienced a decrease in *B. burgdorferi* s.s. prevalence, while two experienced an increase in *B. burgdorferi* s.s. prevalence following management efforts. Our findings demonstrate a weak, non-significant relationship among white-tailed deer density and *B. burgdorferi* s.s. prevalence across a broad geographic range but reveal variable effects on *B. burgdorferi* s.s. prevalence following deer removal.

White-tailed deer play an important role in the reproductive stage of *I. scapularis*, with their large body and home range sizes supporting carriage of large numbers of ticks. Higher deer densities are assumed to facilitate high densities of immature *I. scapularis* in the environment. However, the consequence of high larval and nymphal burden in the landscape on *B. burgdorferi* s.s. transmission risk remains poorly understood. There is a possibility that high densities of immature life stages in the environment will result in larvae and nymphs utilizing a broader range of hosts, including those that do no serve as *B. burgdorferi* s.s. reservoirs such as white-tailed deer (Huang et al., 2019). This may theoretically reduce *B. burgdorferi* s.s. transmission risk to larvae; however, this association has not been made. A more plausible scenario is that higher densities of immature life stages in the landscape result in an increased density of larvae and nymphs found on individual small mammal hosts–such as white-footed mice–which could result in increased risk for transmission of *B. burgdorferi* s.s. among ticks during co-feeding (Levin et al., 1997, Belli et al., 2017) and may increase the risk of horizontal transmission to small mammal hosts (Eisen, 2018). Further, larval and nymphal burdens are often heterogenous among white-footed mouse hosts, with few individuals supporting high tick loads and likely responsible for the majority of *B. burgdorferi* s.s. transmission events (Brunner and Ostfeld, 2008, Devevey and Brisson, 2012).

Conversely, low densities of white-tailed deer should limit the density of immature *I. scapularis* in the landscape; however, it is unclear how this corresponds to reduced *B. burgdorferi* s.s. transmission risk. Reduced densities of immature life stages in the environment may not translate to lowered densities of nymphal and larval ticks on individual white-footed mice and other reservoir hosts, particularly if on-host densities are driven by host behavior (Devevey and Brisson, 2012). Reductions of white-tailed deer populations can aid vegetation regeneration and bolster small mammal communities (Rooney and Waller, 2003, Byman, 2013, Shelton et al., 2014), though diverse host communities may not reduce *B. burgdorferi* s.s. infection risk (no evidence for the dilution effect in some studies; States et al., 2014, Linske et al., 2018). The variable–observations of both increase and decrease–non-significant, effect of deer management on *B. burgdorferi* s.s. prevalence suggests that deer reductions are likely not the sole factor influencing pathogen carriage in nymphs and warrants further investigation.

It is possible that other environmental factors–including climate, weather, and abundance of host species–may play a larger role in *B. burgdorferi* s.s. prevalence than white-tailed deer densities. For example, in similar systems in Europe, the abundance of rodent hosts is strongly correlated with the infection prevalence of certain pathogens in *Ixodes* nymphs (*Borrelia afzelii* and *Neoehrlichia mikurensis*; Krawczyk et al., 2020). Further, host selection may influence *B. burgdorferi* s.s. transmission risk and prevalence in nymphs, though, this has mainly been observed across a north-south gradient (Ginsberg et al., 2021). In this study, host abundance for immature life stages were not available. However, weather data were incorporated. Here, within-year April relative humidity was significantly negatively correlated with *B. burgdorferi* s.s. prevalence; however, the connection between spring relative humidity and *B. burgdorferi* s.s. infection prevalence is unintuitive. Within-year climate and weather variables are not likely to affect acquisition of *B. burgdorferi* s.s. infection by nymphal ticks, as this should occur during the previous year at the time of the first bloodmeal as a larva. Presumably, higher spring humidity facilitates off-host survival of nymphs (Stafford, 1994), but prolonged questing periods are not expected to influence *B. burgdorferi* s.s. infection status (or transmission potential; Samanta et al., 2022). One hypothesis is that there is an interaction between weather and *B. burgdorferi* s.s. infection status that could influence survival, behavior, or detection of *B. burgdorferi* s.s. infected nymphs. For example, *B. burgdorferi* s.s. infected nymphs showed increased phototaxis and tended to quest at increased heights, which may make them more vulnerable to environmental conditions (Lefcort and Durden, 1996). However, evidence for such an interaction is currently lacking and would require additional research. More likely, environmental factors, such as humidity, may be correlated with other biotic factors not assessed here.

### 4.4. Deer density thresholds, DIN, and human Lyme disease risk

Previous research suggested there may be a deer density threshold below which there would be gradual declines in tick abundances (Wilson et al., 1988), with more recent suggestions that this threshold should be 3–5 deer per km^2^ (Telford, 2017). However, there was limited scientific support for this claim. We found variable reductions in the density of infected nymphs–the most representative metric for human risk in the absence of human tick encounter or human case data–for parks reaching deer densities of 20 deer per km^2^, while only one park attained a density of less than 5 deer per km^2^. Specific target densities are useful for managers to strive for but may not represent thresholds, per se, such that reductions that do not reach the target may still be effective (Lloyd-Smith et al., 2005). All parks had fewer than 5 *B. burgdorferi* s.s. infected nymphs per 750 m^2^ at a white-tailed deer density of 20 deer per km^2^, which reduced to 3 or fewer infected nymphs per 750 m^2^ at a deer density of 5 deer per km^2^. However, given the limitations of our data (see below), the efficacy of deer density thresholds needs to be explored further.

Our results show that white-tailed deer density is correlated–though, variably–with nymph densities (as was demonstrated by Deblinger et al., 1993); however, deer density has no clear relationship with *B. burgdorferi* s.s. prevalence. Thus, additional factors not addressed here are likely influencing nymph densities, to some extent, and substantially contributing to *B. burgdorferi* s.s. prevalence. These findings suggest that managing white-tailed deer densities alone may not be effective in reducing DIN, but our interpretation is limited by the nature of our dataset, with few tick samples post-management for some locations. Further, our DIN results were estimated using two data streams–density of nymphs and infection prevalence–which vary in their relationship with deer density. Nevertheless, deer reduction efforts may be useful when implemented in combination with other management actions to target *B. burgdorferi* s.s. prevalence and nymph density, simultaneously. Integrated management regimes are already being tested without a clear consensus on best practices (Williams et al., 2017, Mandli et al., 2021), and we demonstrate that deer reduction may be a useful tool for inclusion in future multi-faceted management regimes.

### 4.5. Limitations

Our study documents a regional scale assessment of the efficacy of white-tailed deer management on controlling nymph densities in the environment, and while these findings contribute additional understanding of this complex topic to the literature, they are not without limitations. Most notably, across our study sites and years, we had only one sampling point where white-tailed deer density reached ≤5 deer per km^2^. While this was a limitation for the lowest end of the white-tailed deer density range, there were ~20 data points with corresponding deer densities between 5–10 deer per km^2^, and thus, we believe our model projections to be robust, even at the lowest end of this range. However, additional data at densities ≤5 deer per km^2^ will inform variability at this lower end. Another caveat of our research was that post-management data were limited for some parks that engaged in deer reductions. For three of the five parks, management was recently initiated–in 2018 or 2019–and only 2–3 years of post-management data were available. Further, low nymph densities, which were likely a result of deer removal efforts, limited our sample sizes for pathogen testing post-management. Continued surveillance in these parks will allow for a long-term assessment of the efficacy of deer management practices.

## 5. Conclusions

White-tailed deer management and density thresholds are often cited as effective methods for mitigating human Lyme disease risk. We demonstrate that deer density is positively associated with *I. scapularis* nymph densities across a broad geographic scale but that meeting stringent deer density thresholds alone may not result in large declines in the density of host-seeking infected nymphs (DIN). Instead, deer management may be utilized as part of an integrated strategy for reducing DIN. While this research expands our understanding of the influence of white-tailed deer density on DIN, there is still a need for human case data to explicitly link deer density to human Lyme disease risk. Future work should aim to assess this relationship with human Lyme disease data that minimize uncertainties, such as uncertainty in exposure location. Potential opportunities may include utilizing worker’s compensation claims from occupations that spend time in tick habitat (e.g., park employees).

## Supplementary Material

mmc2

mmc3

mmc4

mmc5

mmc1

## Figures and Tables

**Table 1:** National parks that engaged in *Ixodes scapularis* nymph spring surveillance from 2014–2022. Park names, park IDs, location by state or region (“State”), number of 750 m^2^ transects per park and region (“T”), maximum density of nymphs observed (“Max no.”), and the number of nymphs tested (“Tested”) and positive for *Borrelia burgdorferi* sensu stricto (“Bbss+”) each year are presented.

Park Name	Park ID	State	T (Region)	2014		2015		2016		2017		2018		2019		2020		2021		2022	
				Max no.	Tested (Bbss+)	Max no.	Tested (Bbss+)	Max no.	Tested (Bbss+)	Max no.	Tested (Bbss+)	Max no.	Tested (Bbss+)	Max no.	Tested (Bbss+)	Max no.	Tested (Bbss+)	Max no.	Tested (Bbss+)	Max no.	Tested (Bbss+)
Catoctin Mountain Park	CATO	MD	1	0	10 (3)	2	3 (1)	2	n/a			4	5 (0)	4	4 (0)	7	7 (1)	5	10 (1)	35	43 (2)
			2	26	48 (11)	10	19 (7)	21	n/a			8	9 (2)	29	27 (3)	15	7 (2)	12	22 (3)	17	32 (2)
			3	13	45 (6)	19	23 (8)	56	n/a			9	14 (6)	9	5 (0)	5	5 (0)	20	24 (3)	17	21 (6)
Chesapeake and Ohio Canal National Historic Park	CHOH	MD	1							9	n/a	2	3 (0)	2	2 (0)	4	17 (0)	4	5 (0)	8	12 (0)
			2							6	n/a	4	5 (0)	4	4 (0)	18	24 (0)	8	10 (0)	3	6 (0)
			3							6	n/a	7	12 (1)	4	4 (0)	1	2 (0)	6	6 (0)	2	2 (1)
Fire Island National Seashore	FIIS	NY	1 (WFE)	98	50 (12)	90	50 (5)	56	n/a			76	n/a	38	20 (2)	37	n/a	60	n/a	10	n/a
			2 (WFE)	18	49 (4)	61	50 (9)	49	n/a												
			3 (WFE)	80	50 (6)	153	50 (14)	86	n/a			114	n/a	45	14 (4)	45	47 (10)	48	n/a	15	n/a
			4 (DP)									22	n/a	3	n/a	13	14 (5)	29	n/a	12	n/a
			5 (SH)									74	n/a	46	23 (8)	37	37 (14)	91	n/a	113	n/a
			6 (WA)									0	n/a	0	n/a	0	n/a	0	n/a	0	n/a
			7 (WA)									0	n/a	0	n/a	0	n/a	0	n/a	0	n/a
			8 (SH)									0	n/a	0	n/a			0	n/a	0	n/a
			9 (WA)									0	n/a	0	n/a	0	n/a	0	n/a	0	n/a
Gettysburg National Military Park	GETT	PA	1	10	16 (0)	20	25 (2)											12	19 (6)	0	n/a
			2	30	30 (9)	5	6 (2)											32	58 (13)	51	51 (15)
			3	17	17 (7)	2	20 (4)											15	19 (3)	5	8 (0)
Manassas National Battlefield	MANA	VA	1	23	34 (6)	10	18 (7)					0	n/a	3	n/a	6	n/a	0	n/a	5	5 (2)
			2	11	36 (7)	12	19 (5)							5	1 (0)	24	3 (0)	1	1 (1)	6	6 (2)
			3	72	50 (7)	55	50 (9)					5	n/a	5	6 (2)	12	12 (0)	2	4 (0)	4	7 (0)
			4									6	n/a								
Monocacy National Battlefield	MONO	MD	1	9	10 (1)	7	7 (2)											2	4 (0)	8	10 (3)
			2			3	3 (0)														
Prince William Forest Park	PRWI	VA	1	11	32 (1)	15	26 (0)					3	2 (0)	2	2 (0)	10	40 (0)	5	9 (1)	4	4 (0)
			2	15	23 (1)	10	20 (1)					0	2 (0)	0	n/a	6	6 (0)	2	2 (0)	1	1 (0)
			3	11	24 (2)	10	16 (1)					0	n/a	2	n/a	2	2 (0)	3	4 (0)	1	1 (0)
Rock Creek National Park	ROCR	DC	1	91	50 (16)	27	37 (10)	3	n/a			7	17 (0)	8	9 (0)	10	15 (1)	4	4 (0)	17	19 (0)
			2	58	55 (12)	10	11 (5)	2	n/a			5	22 (1)	5	4 (0)	6	5 (0)	1	1 (0)	6	6 (0)
			3	146	50 (14)	71	50 (5)	10	n/a			32	37 (6)	10	13 (1)	24	38 (1)	6	10 (1)	2	4 (1)

**Table 2:** Response and predictor variables for the four models developed to understand the effect of white-tailed deer density and management on *Ixodes scapularis* density and *Borrelia burgdorferi* sensu stricto infection prevalence. The “term” reflects how each variable is displayed in the model, “type” describes whether each term is a response or predictor variable, and “description” briefly defines each (see Methods for more details). Models 01 through 04 are marked by an ‘x’ if the term is included in each respective model and “RE” and “P” designate whether the variable was a random effect or predictor, respectively.

Term	Type	Description	*Model*			
			01	02	03	04
density	Response	Density of *Ixodes scapularis* nymphs per 750 m^2^	x	x		
Bbss_pos | Bbss_neg	Response	Proportion of *Borrelia burgdorferi* sensu stricto positive *I. scapularis* nymphs to *Borrelia burgdorferi* sensu stricto negative nymphs			x	x
pHDD	Predictor	Proportion of hot-dry-days	x	x		
RH_April	Predictor	April relative humidity	x	x	x	x
RH_May	Predictor	May relative humidity			x	x
PZ_May	Predictor	May Palmer’s Z-Index			x	x
Park_ID	Predictor or Random effect	Park identity	x (RE)	x (P)	x (RE)	x (P)
deer_dens	Predictor	Yearly deer density per km^2^	x		x	
deer_manage	Predictor	Deer management (binary) with a value of 0 if no deer reductions occurred two or more years prior, and a value of 1 if deer reduction did occur two or more years prior		x		x

**Table 3:** The predicted effects of white-tailed deer reductions on *Ixodes scapularis* nymph densities, *Borrelia burgdorferi* sensu stricto infection prevalence, and density of *B. burgdorferi* s.s.-infected nymphs at five United States national parks that engaged in deer reduction management. The number of years with deer and nymph density data pre- and post- management efforts are shown, with their corresponding years in parentheses. Deer density data were available from 2012–2020 and nymph density data were collected from 2014–2022. Discrepancies in the number of years with available data relative to the data collection periods indicate missing data (see Table 1). Model predictions of the mean and 95% credible interval (95% CI; in brackets) were made for nymph density per 750 m^2^ (“Mean nymph density”), *Borrelia burgdorferi* prevalence s.s. (“Mean Bbss prev.”), and density of infected nymphs (“Mean DIN”) for each park before (“pre”) and following (2-years lagged; “post”) deer reduction management. Note, FIIS (WFE) did not have *B. burgdorferi* s.s. prevalence data available post-management. The percent change in mean DIN was estimated using model predicted DIN means for pre- and post- management.

Park ID (Region)	No. of mgmt. years	No. years with deer density datapre- / post-(Year range)	No. years with nymph density datapre- / post-(Year range)	Mean deer density pre	Mean deer density post	Total deer removed (Avg. per year)	Mean nymph density pre [95% CI]	Mean nymph density post [95% CI]	Mean Bbss prev. pre [95% CI]	Mean Bbss prev. post [95% CI]	Mean DIN pre [95% CI]	Mean DIN post [95% CI]	Decline in DIN
ROCR	8	1 / 8 (2012 / 2013–20)	1 / 7 (2014 / 2015–22)	28.3	13.5	505 (56)	58 [3, 206]	14 [0, 47];	0.17 [0.07, 0.30]	0.09 [0.03, 0.16	9.38 [0.26, 37.24]	1.26 [0.00, 4.94]	86.6%
MONO	5	4 / 5 (2012–15 / 2016–20)	2 / 2 (2014–17 / 2018–22)	73.6	33.9	659 (110)	9 [0, 37],	6 [0, 29]	0.10 [0.02, 0.25]	0.22 [0.04, 0.49]	0.86 [0.00, 4.08]	1.59 [0.00, 8.74]	−84.9
CHOH	3	6 / 3 (2012–17 / 2018–20)	3 / 3 (2014–19 / 2020–22)	31.6	21.4	158 (40)	5 [0, 19],	6 [0, 24]	0.04 [0, 0.14]	0.01 [0, 0.06]	0.21 [0.00, 1.12]	0.09 [0.00, 0.60]	57.1%
MANA	3	6 / 2 (2012–17 / 2018–20)	4 / 3 (2014–19 / 2020–22)	31.6	11.4	451 (150)	16 [0, 55]	9 [0, 34]	0.12 [0.05, 0.22]	0.17 [0.04, 0.35]	1.99 [0.06, 7.84]	1.71 [0.00, 7.67]	14.1%
FIIS (WFE)	2	4 / 2 (2012–18 / 2019–20)	6 / 2 (2014–20 / 2021–22)	51.0	46.6	259 (65)	76 [4, 256]	48 [4, 180]	–	–	–	–	–

## Data Availability

Data are available from Martin et al. (2023; doi.org/10.5066/P9LSI8K9).

## Abbreviations

DIN: density of infected nymphs
