# Supplementary material for: Deer management generally reduces densities of nymphal Ixodes scapularis, but not prevalence of infection with Borrelia burgdorferi sensu stricto
Source: Ticks Tick Borne Dis. Author manuscript; Available in PMC 2024 Jul 23. (PMC11265508; doi:10.1016/j.ttbdis.2023.102202)
Supplement: mmc1 [file NIHMS2010120-supplement-mmc1.docx]

Supplementary Material for Deer management reduces nymph tick densities but not pathogen prevalence

Authors: AM Martin, D Buttke, J Raphael, K Taylor, HS Ginsberg, PC Cross

Journal: Submitted to Ticks and Tick-Borne Diseases

DOI: TBD

# TABLE OF CONTENTS:

| Title and description | Page number |
| --- | --- |
| **Supplementary Material I.** National park names, IDs, transect locations, and climate information corresponding to tick surveillance that occurred between 2014–2022. | 2 |
| **Supplementary Material II**. National parks deer density estimates from 2012–2020. | 3 |
| **Supplementary Material III**. Deer removal efforts at eight national parks (and within park regions) from 2012–2020. | 4 |
| **Supplementary Material IV**. Pathogen model results (model 03) when only years with N≥10 Ixodes scapularis nymph ticks were submitted for pathogen testing. | 5 |
| **Supplementary Material V**. Model 01 intercept estimates for the random effect of national park. | 6 |
| **Supplementary Material VI**. Model results and predictions assessing the effect of deer management on *Ixodes scapularis* nymph tick densities and *Borrelia burgdorferi* prevalence. | 7 |
| **Supplementary Material VII.** Model 01 R code. | R file |
| **Supplementary Material VIII.** Model 02 R code. | R file |
| **Supplementary Material IX.** Model 03 R code. | R file |
| **Supplementary Material X.** Model 04 R code. | R file |

*Any use of trade, firm, or product names is for descriptive purposes only and does not imply endorsement by the U.S. Government.*

# Supplementary Material I

| **Park name** | **Park ID** | **State** | **Transects** | **Start Latitude** | **Start Longitude** | **NOAA station**  **[Euclidean distance**  **from park center]** | **CONUS Climate Division** |
| --- | --- | --- | --- | --- | --- | --- | --- |
| Catoctin Mountain Park | CATO | MD | 1  2  3 | 39.64665  39.66061  39.67250 | -77.44190  -77.48267  -77.49668 | USC00182906 [15.7 km] | 1806 |
| Chesapeake and Ohio Canal  National Historic Park | CHOH | MD | 1  2  3 | 38.99977  38.99293  38.98479 | -77.24808  -77.23932  -77.23076 | USC00182325 [11.5 km] USC00182336 (2022) [30.2 km] | 1803 |
| Fire Island National Seashore | FIIS | NY | 1 (WFE)  2 (WFE)  3 (WFE)  4 (n/a)  5 (SH)  6 (WA)  7 (WA)  8 (SH)  9 (WA) | 40.77237  40.77134  40.76492  40.68540  40.65570  40.73930  40.73406  40.65404  40.70673 | -72.82810  -72.81701  -72.82144  -73.00400  -73.11210  -72.84477  -72.86465  -73.11496  -72.94653 | USW00004781 [13.0 km] | 3004 |
| Gettysburg National  Military Park | GETT | PA | 1  2  3 | 39.83140  39.78464  39.79056 | -77.25237  -77.24969  -77.24048 | USC00363226 [12.0 km] | 4204 |
| Manassas National Battlefield | MANA | VA | 1  2  3  4 | 38.80522  38.82619  38.82771  38.81320 | -77.56005  -77.54812  -77.50881  -77.52190 | USC00445204 [10.8 km] | 4404 |
| Monocacy National Battlefield | MONO | MD | 1  2 | 39.36133  39.35889 | -77.40165  -77.39158 | USC00182336 [17.9 km] USW00093738 (2013) [46.6 km] | 1806 |
| Prince William Forest Park | PRWI | VA | 1  2  3 | 38.60422  38.58063  38.58213 | -77.40400  -77.39269  -77.37242 | USW00013773 [11.5 km] | 4404 |
| Rock Creek National Park | ROCR | DC | 1  2  3 | 38.95688  38.98547  38.96021 | -77.05172  -77.05262  -77.05164 | USC00182325 [10.4 km]  USC00182336 (2022) [36.7 km] | 1804 |

**Supplementary Material I**. National park names, IDs, transect locations, and climate information corresponding to tick surveillance that occurred between 2014–2022. FIIS deer density regions are listed in parentheses next to the transect number.

# Supplementary Material II

**Supplementary Material II**. National parks deer density estimates from 2012–2020. Deer density data are available at the park scale for all parks and at within-park regional scale for FIIS.

| Park ID (region) | 2012 | 2013 | 2014 | 2015 | 2016 | 2017 | 2018 | 2019 | 2020 |
| --- | --- | --- | --- | --- | --- | --- | --- | --- | --- |
| CATO | 7.08 | 17.01 | 13.60 | 8.01 | 11.96 | 9.00 | 9.26 | 6.37 | 12.22 |
| CHOH | 30.02 | 26.42 | 25.81 | 57.26 | 28.80 | 20.96 | 29.89 | 28.62 | 5.79 |
| FIIS (SH) | 63.00 | 45.98 | 43.14 | 49.62 | 47.68 | 30.00 | 10.50 | 38.10 | 9.90 |
| FIIS (WA) | n/a | 27.23 | n/a | n/a | 30.60 | 27.90 | 22.90 | 12.50 | 6.60 |
| FIIS (WFE) | 35.80 | n/a | n/a | 44.10 | n/a | 49.90 | 74.30 | 62.70 | 28.40 |
| GETT | 12.52 | n/a | 11.77 | 7.41 | 6.62 | 5.90 | 15.79 | 5.80 | n/a |
| MANA | 33.99 | 34.35 | 29.02 | 38.24 | 23.85 | 30.13 | n/a | 15.69 | 7.17 |
| MONO | 81.78 | 71.58 | 74.84 | 66.29 | 66.14 | 36.70 | 27.26 | 22.70 | 16.89 |
| PRWI | 17.10 | 23.77 | 5.91 | 15.02 | 13.52 | 16.37 | 12.23 | 10.14 | 6.52 |
| ROCR | 28.34 | 30.05 | 15.46 | 7.41 | 7.59 | 21.39 | 15.11 | 7.33 | 3.47 |

# Supplementary Material III

**Supplementary Material III**. Deer removal efforts at eight national parks (and within park regions) from 2012–2020. Park and park subregion IDs are ordered by deer removal efforts for parks (based on years of management; highest first) and based on whether there are pre- and post- nymph density data. The number of deer removed is reported by park for each year. The total number of years (“Total years”) that deer were removed is summarized and “Total removed” is the sum of the number of deer removed across years within each park, with the average yearly number removed in parenthesis (rounded). Mean deer density pre-management (“Mean deer density pre”) and mean deer density post-management (lagged 2-years; “Mean deer density post”) are presented (per km^2^), with minimum and maximum densities in parentheses. Parks below the black line did not have at least two years of tick density data pre- and post- management (lagged) and were not included in the analyses assessing the effect of binary deer management.

| Park ID | Park Area | 2012 | 2013 | 2014 | 2015 | 2016 | 2017 | 2018 | 2019 | 2020 | Total years | Total removed | Mean deer density pre  (minimum, maximum) | Mean deer density post  (minimum, maximum) |
| --- | --- | --- | --- | --- | --- | --- | --- | --- | --- | --- | --- | --- | --- | --- |
| ROCR | 7.1 | 0 | 20 | 161 | 26 | 36 | 94 | 83 | 34 | 24 | 8 | 505 (56) | 28.34 (28.34, 28.34) | 13.48 (3.47, 30.05) |
| MONO | 6.07 | 0 | 0 | 0 | 0 | 264 | 134 | 79 | 76 | 44 | 5 | 659 (110) | 73.62 (66.29, 81.78) | 33.94 (16.89, 66.14) |
| CHOH | n/a | 0 | 0 | 0 | 0 | 0 | 0 | 20 | 74 | 12 | 3 | 158 (40) | 31.55 (20.96, 57.26) | 21.43 (5.79, 29.89) |
| MANA | 18.3 | 0 | 0 | 0 | 0 | 0 | 0 | 241 | 116 | 94 | 3 | 451 (150) | 31.60 (23.85, 38.24) | 11.43 (7.17, 15.69) |
| FIIS-WFE | n/a | 0 | 0 | 0 | 0 | 0 | 0 | 0 | 25 | 130 | 2 | 259 (65) | 51.03 (35.80, 74.3) | 45.55 (28.4, 62.7) |
| FIIS SH | n/a | 0 | 0 | 0 | 0 | 0 | 0 | 0 | 0 | 20 | 1 | 20 | 41.00 (10.5, 63.00) | 9.90 (9.90, 9.90) |
| FIIS WA | n/a | 0 | 0 | 0 | 0 | 0 | 0 | 0 | 0 | 2 | 1 | 2 | 24.23 (12.5, 30.6) | 6.6 (6.60, 6.60) |
| CATO | 24.9 | 226 | 161 | 156 | 119 | 76 | 105 | 72 | 69 | 13 | 9 | 997 (111) | n/a | 10.50 (6.37, 17.01) |
| GETT | 24.2 | 180 | 224 | 149 | 132 | 180 | 250 | 200 | 300 | 250 | 9 | 1865 (207) | n/a | 9.40 (5.78, 15.79) |
| PRWI | 65.1 | 0 | 0 | 0 | 0 | 0 | 0 | 0 | 0 | 0 | 0 | 0 | 13.40 (5.91, 23.77)  (no deer management) | n/a |

# Supplementary Material IV

**Supplementary Material IV**. Pathogen model results (model 03) when only years with N>1 *Ixodes scapularis* nymph ticks were submitted for pathogen testing (A-C) and when N≥10 (D-F). (**A, D**) Coefficient plots for model 03 assessing the impact of deer density on *Borrelia burgdorferi* infection prevalence in nymph *I. scapularis* (mean point estimate, 50% mass interval, and 95% mass interval). (**B, E**) Mean predicted prevalence of *B. burgdorferi* s.s. infection in nymphs across a range of deer densities (0–85 per km^2^) extrapolated to the maximum deer density observed at each park. (**C, E**) Model 03 intercept estimates and 95% credible intervals for the random effect of national park in order of decreasing value. The intercept and 95% posterior credible intervals for the random effect of national park when N>1 (plot C) in order of decreasing value: GETT 0.60 [95% PCI = -0.27, 1.49]; FIIS 0.47 [95% PCI = -0.34, 1.30]; CATO 0.45 [95% PCI = -0.37, 1.26]; MANA 0.40 [95% PCI = -0.42, 1.21]; ROCR 0.26 [95% PCI = -0.53, 1.06]; MONO 0.15 [95% PCI = -0.95, 1.21]; PRWI -1.35 [95% PCI = -2.46, -0.47]; and CHOH -1.47 [95% PCI = -2.92, -0.46].

| **A** (N>1) Model variable coefficients | **B** (N>1) Predicted prevalence of *B. burgdorferi* s.s | **C** (N>1) Random effect |
| --- | --- | --- |
| 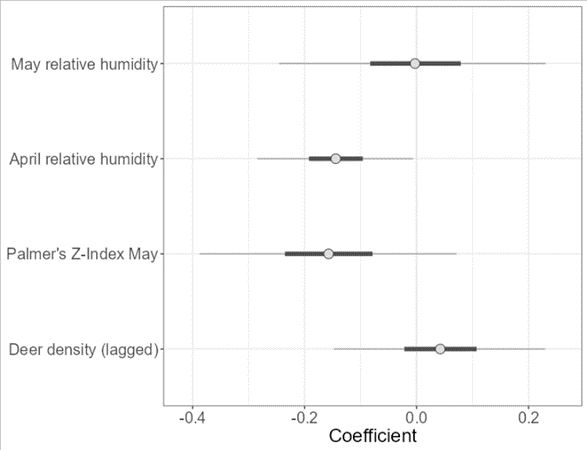 | 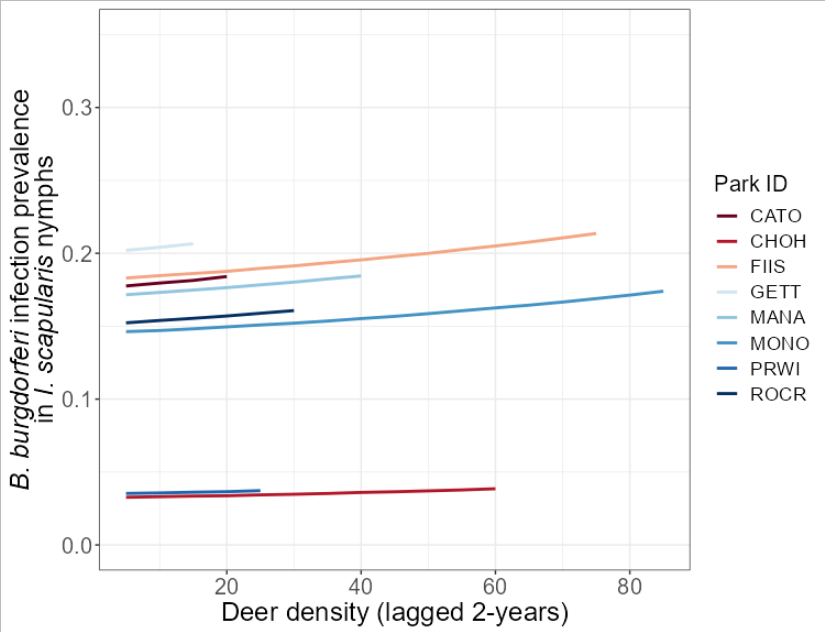 | 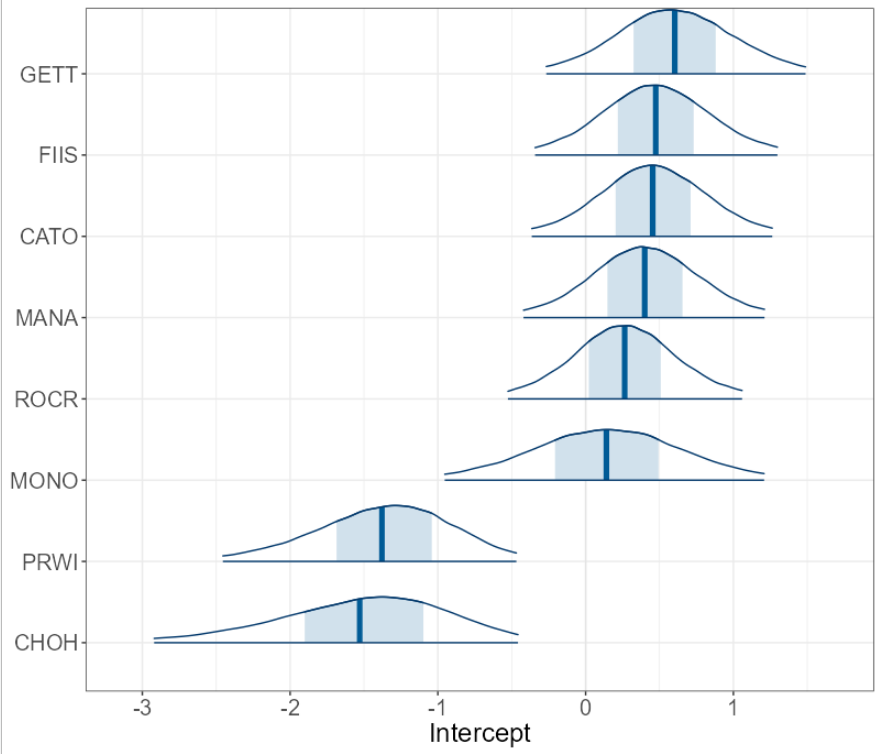 |
| **D** (N≥10) Model variable coefficients | **E** (N≥10) Predicted prevalence of *B. burgdorferi* s.s | **F** (N≥10) Random effect |
| 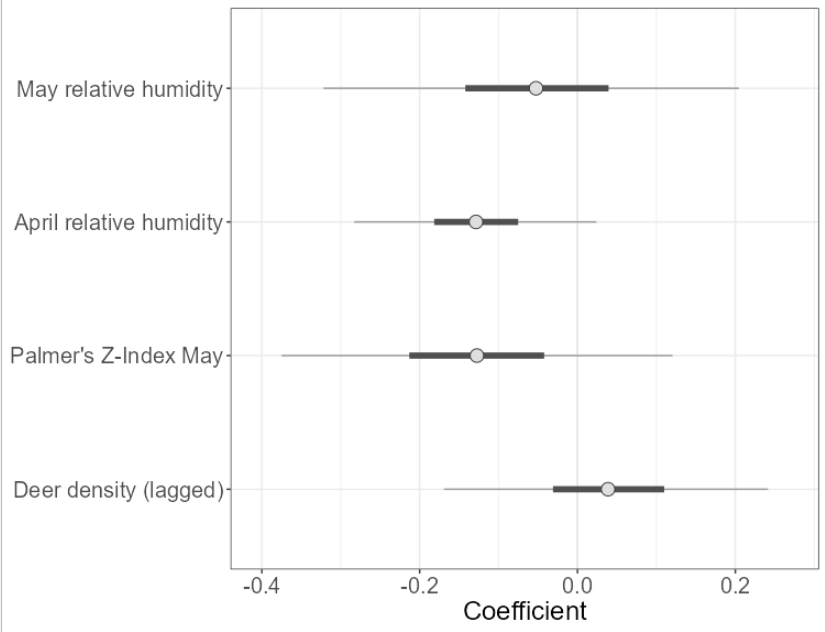 | 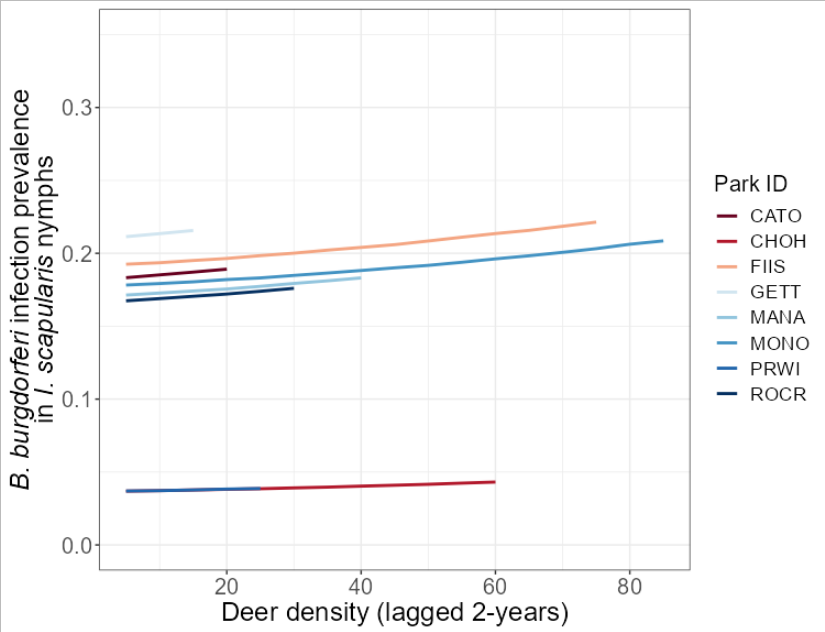 | 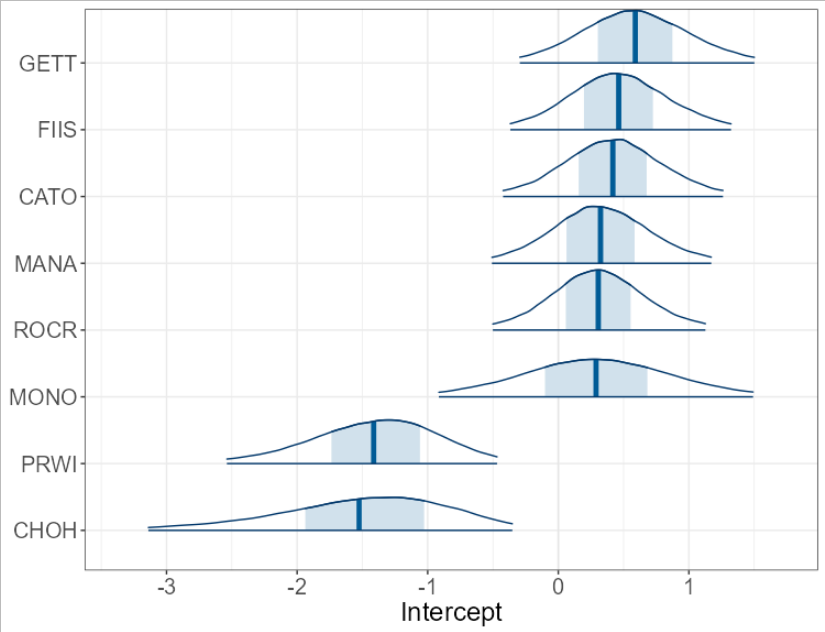 |

# Supplementary Material V

**Supplementary Material V**. Model 01 intercept estimates for the random effect of national park. In order of decreasing value, the intercept and 95% posterior credible intervals are: GETT 0.70 [95% PCI = -0.13, 1.81]; ROCR 0.61 [95% PCI = -0.06, 1.42]; CATO 0.54 [95% PCI = -0.14, 1.38]; FIIS 0.40 [95% PCI = -0.35, 1.25]; MANA 0.030 [95% PCI = -0.73, 0.85]; PRWI -0.56 [95% PCI = -1.33, 0.21]; MONO -0.68 [95% PCI = -1.88, 0.37]; CHOH -0.71 [95% PCI = -1.51, 0.05]


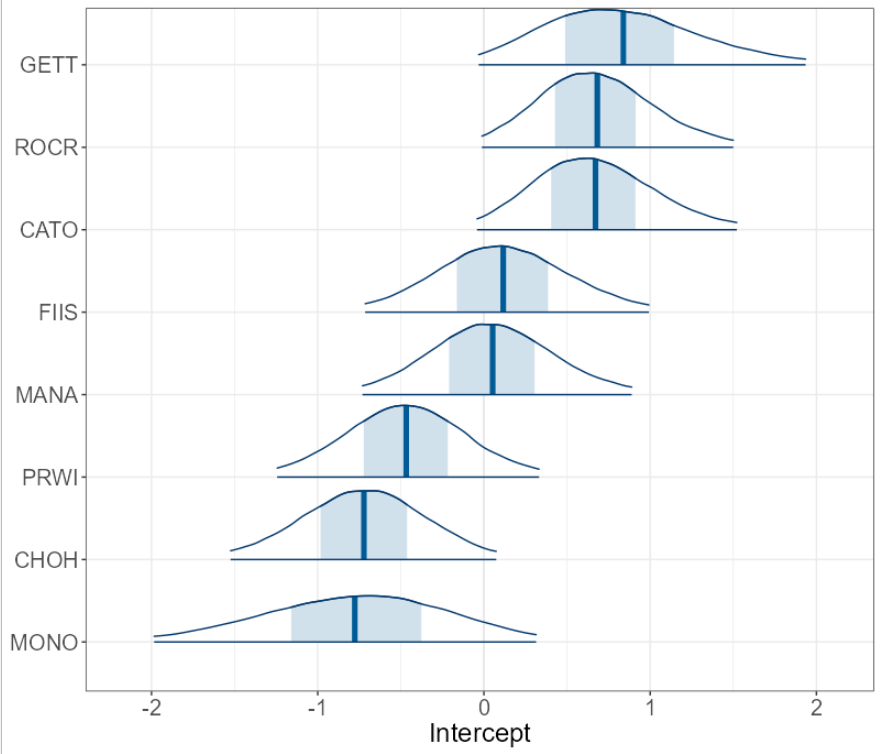


# Supplementary Material VI

**Supplementary Material VI**. Model results and predictions assessing the effect of deer management on *Ixodes scapularis* nymph tick densities and *Borrelia burgdorferi* prevalence. (A) Coefficient plot for model 02, investigating the impact of binary deer management on nymph tick densities. The plot shows the mean point estimate, the 50% probability mass interval (thick, grey line), and the 95% probability mass interval (thin, grey line). The coefficient values for the interaction between deer management and park ID are relative to the reference park (ROCR, intercept and management interaction). (B) Nymph tick density predictions under no deer management (0) and deer reduction practices (1) using model 01 results. Mean predicted values are presented by the solid points and the 95% credible intervals represented by the vertical lines. Raw data are shown by the semi-transparent points and both predicted means and raw data are colored according to park ID. (C) The coefficient plot for model 04 assessing the impact of deer management on B. burgdorferi infection in nymph I. scapularis (mean point estimate, 50% mass interval, and 95% mass interval). (D) Mean predicted prevalence of infected nymphs (with 95% credible intervals) under scenarios of no deer management (0) and deer reduction (2-years lagged; 1).

| **A** | **B** |
| --- | --- |
| 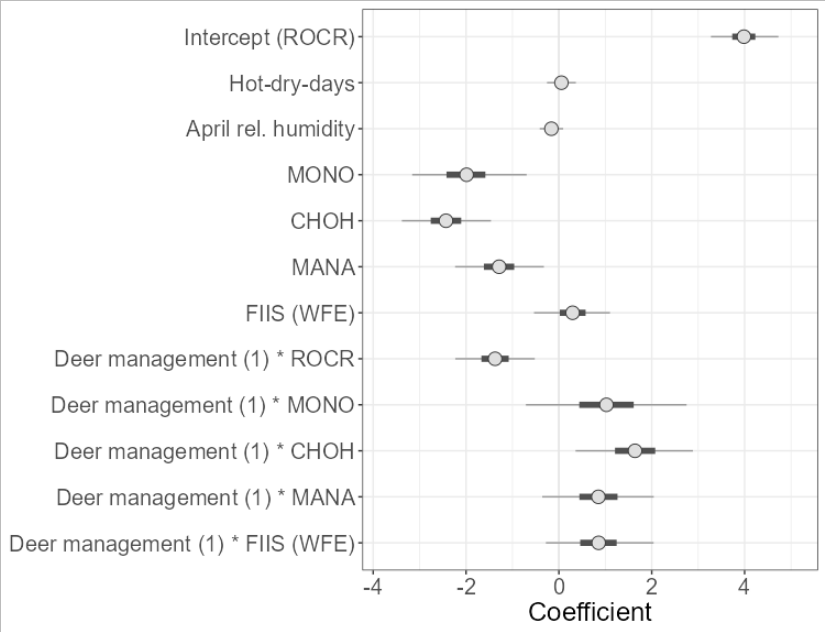 | 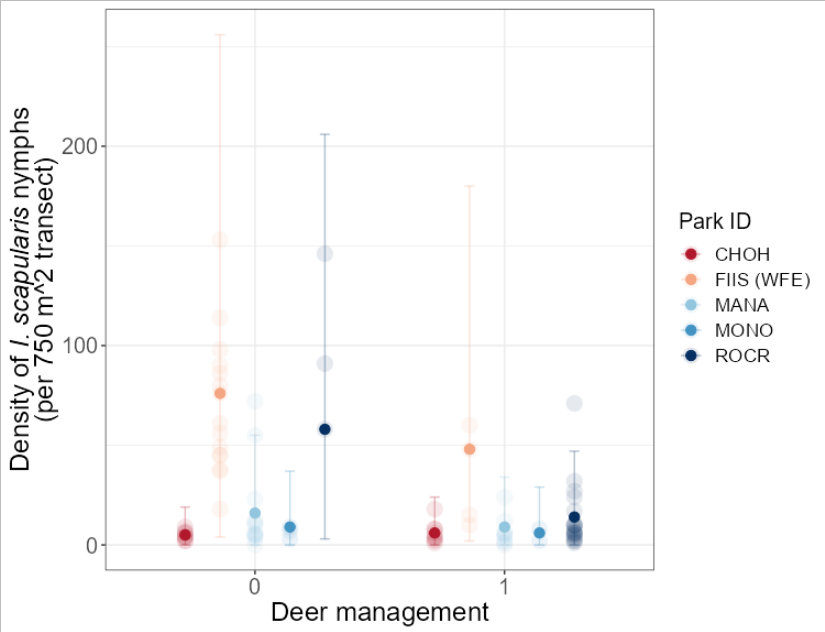 |
| **C** | **D** |
| 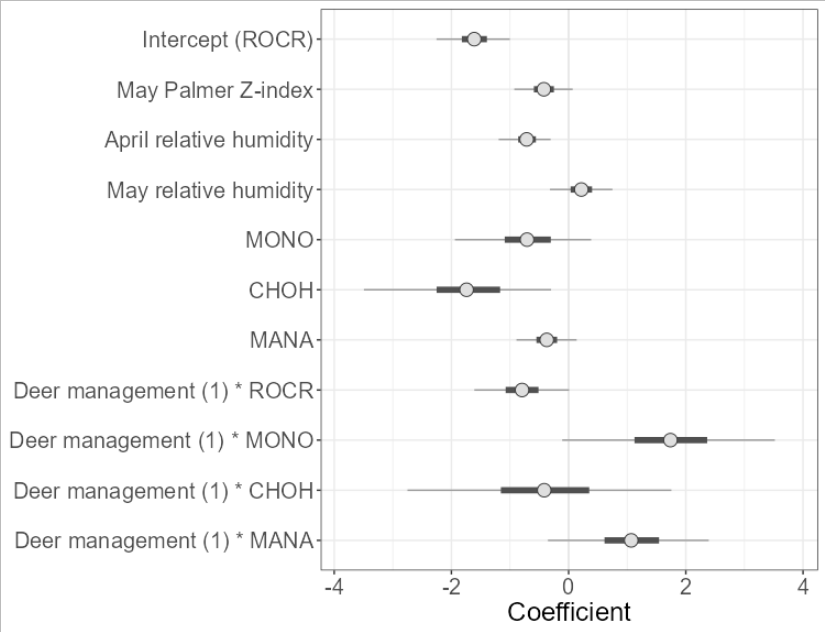 | 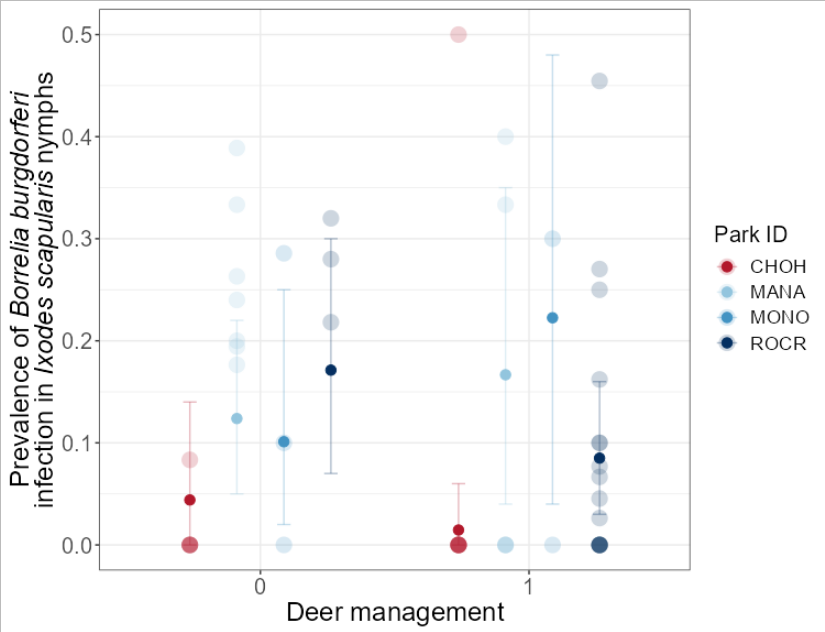 |
